# Analysing data from the psycholinguistic visual-world paradigm: Comparison of different analysis methods

**DOI:** 10.3758/s13428-022-01969-3

**Published:** 2022-11-17

**Authors:** Aine Ito, Pia Knoeferle

**Affiliations:** 1 Department of German Studies and Linguistics, Humboldt-Universität zu Berlin, Berlin, Germany; 2 Department of English, Linguistics and Theatre Studies, National University of Singapore, Block AS5, 7 Arts Link, Singapore, 117570 Singapore; 3 Berlin School of Mind and Brain, Berlin, Germany; 4 Einstein Center for Neurosciences Berlin, Berlin, Germany

**Keywords:** Visual-world paradigm, Eye-tracking, ANOVA, Linear mixed-effects model, Growth curve analysis, Cluster-based permutation analysis, Bootstrapped differences of timeseries, Generalised additive modelling, Divergence point analysis

## Abstract

In this paper, we discuss key characteristics and typical experimental designs of the visual-world paradigm and compare different methods of analysing eye-movement data. We discuss the nature of the eye-movement data from a visual-world study and provide data analysis tutorials on ANOVA, *t*-tests, linear mixed-effects model, growth curve analysis, cluster-based permutation analysis, bootstrapped differences of timeseries, generalised additive modelling, and divergence point analysis to enable psycholinguists to apply each analytical method to their own data. We discuss advantages and disadvantages of each method and offer recommendations about how to select an appropriate method depending on the research question and the experimental design.

## Introduction

The visual-world paradigm (VWP) has been widely used to investigate language processing. This paradigm involves tracking participants’ eye movements with an eye-tracker as they listen to individual sounds, words or sentences and inspect either things in the real world or visual information on a computer display (Huettig et al., 2011; Knoeferle & Guerra, 2016; Salverda & Tanenhaus, 2017). We distinguish a range of eye-movement types (see Rayner, 1998, 2009 for comprehensive reviews) in the VWP (see Pyykkönen-Klauck & Crocker, 2016), among them are fixations (the eye rests), saccades (the eye jumps from an old to a new location) and saccade latencies (Altmann & Kamide, 2004, Appendix on comparative analyses). So-called linking hypotheses (Just & Carpenter, 1980; Magnuson, 2019; Pyykkönen-Klauck & Crocker, 2016; Tanenhaus et al., 2000) permit researchers to interpret these sorts of eye movements as indicative of the cognitive operations implicated in language processing. For example, if listeners hear *the woman,* and they next look more at a nearby woman than man, we can infer from this difference in fixations that they have understood *woman* and established reference to her. Due to its high temporal resolution, the eye-tracking VWP data can provide insight into listeners’ cognitive operations while individual spoken words or entire sentences are unfolding moment-by-moment.

The analysis of fixation data is not without challenges. First, fixation data are binary (a listener looks at an object or not), and as a result they do not meet the assumption of Gaussian-distributed data. Second, they often do not meet assumptions for parametric tests^1^. One of these assumptions is independence of the observations: If a listener makes a fixation on a woman, they necessarily cannot at the same time look at a nearby man, a characteristic that violates the assumption of independence when comparing looks to these two characters. Further, consecutive eye movements are not independent, and where one looks at the onset of *woman* may affect (above and beyond the effects of any manipulations) the location of the next fixation (Barr et al., 2011). Third, and related to the second point, the analysis of fixations across time brings with it issues regarding temporal dependence of consecutive data points. These data characteristics should be taken into consideration when choosing inferential analyses^2^. Several different analysis methods have been proposed for analysing eye-movement data. Considering these options, researchers must select an analysis that is suitable for their research question and the type of data.

The goal of our paper is to provide linguists and psycholinguists (who may not have used the VWP) tutorials on several analysis methods. We compare different analysis methods using data sets from our previous studies and offer recommendations for how to select an analysis method. To this end, we describe typical experimental designs^3^ and variables that should be considered when making analytic decisions (section Design, visual and speech stimuli, timing, interest periods and task; our aim here is not to provide an exhaustive overview of VWP studies; for a more comprehensive overview, see Huettig et al., 2011; and for a shorter overview, see Supplementary file S1 from https://osf.io/tzn8u/). We then describe two data sets (Experiment 1a in Knoeferle and Crocker (2006) and data from Ito et al. (2018b) discussed below, section Descriptions of our data sets). In section Analysis of fixation averages for individual interest periods, we present examples of applying statistical tests such as *t*-tests, analyses of variance (ANOVA), or linear mixed-effects models (LME) for analysing eye-movement data averages. Although some of these analyses (e.g., ANOVAs or *t*-tests) are no longer frequently used to analyse VWP data, we included them as they have been used in published articles, can serve as a first step into the analysis of VWP data for beginners, and often appear as one step in some of the time-course analyses we show in this manuscript (see below). In section The temporal emergence of an effect, we present examples of time-course analyses. We discuss the nature of the time-course data in VWP studies and the hurdles of testing finer-grained differences in the emergence of an effect over time using *t*-tests/ANOVA or LMEs. We also present examples of five alternative approaches (growth curve analysis, cluster-based permutation analysis, bootstrapped differences of timeseries, generalised additive modelling and divergence point analysis) and discuss the advantages and disadvantages of each method.

### Design, visual and speech stimuli, timing, interest periods and task

In a standard VWP setup, participants’ eye movements are measured while they view some objects or printed words and listen to speech simultaneously. Numerous studies have found a link between the auditorily presented linguistic input and eye movements. One of the major advantages of the VWP is that researchers can manipulate both linguistic characteristics (e.g., semantic, syntactic and phonological relationships on both lexical and sentence levels) and participant characteristics (e.g., infants vs adults, young vs older adults, healthy older adults vs patients, native vs non-native speakers).

Below, we lay out decisions that researchers need to make regarding the experimental design and discuss considerations for making the decisions, with the focus on decisions that can affect the analyses. The decisions about the design include what variables to manipulate (also dubbed ‘independent variables’ or ‘predictors’) and what variables to analyse (also termed ‘dependent variables’ or ‘outcomes’) (cf. Glossary). Additional decisions in setting up the experiment concern other design-related issues (e.g., the assignment of item–condition combinations to lists and counterbalancing), the stimuli and their timing (e.g., the type of visual input, i.e., scene vs Ersatz-scene, see Henderson & Ferreira, 2004; timing: static vs dynamic scenes and the scene preview time, see de Almeida et al., 2019; speech rate and the spacing of words, see Andersson et al., 2011), and choice of interest period (time window for the analysis) (see Huettig et al., 2011).

**Table:** 

Glossary	
**Eye-tracking/VWP terms** **Competitor design:** a design that uses a competitor object. The competitor shares certain (e.g. phonological, semantic) representations with the target (the mentioned object). Biased looks to the competitor are used as estimates for the degree of activation of the shared representations. **Fixation:** a period during which the eye gaze is stable on a single location and visual information is processed. **Fixation proportion:** used synonymously with ‘fixation probability’. The proportion of trials in which the fixation falls in an interest area, or the proportion of time spent fixating an interest area within a time window (which is then averaged across trials). **Inspection:** used synonymously with ‘gaze’. The sum of the durations of consecutive fixations to an interest area. **Interest area (or AoI; area of interest):** a predefined area that is used to assess whether a particular object was fixated. It is often a rectangle surrounding the object. Fixations in the interest area containing the target object will be regarded as fixations on the target object. **Interest period:** a predefined time window in which the effect of experimental condition is expected to occur. Eye-movement data in the interest period are used for the analysis. **Saccade:** a rapid, simultaneous eye movement of both eyes between fixations. **Saccade latency:** the time taken to launch a saccade to an interest area relative to a time point of interest (e.g., the onset of the target word). **Sampling rate:** the frequency with which a data point is recorded. For example, if the sampling rate is 500 Hz, a data point is recorded every 2 (= 1000/500) ms. **Target-absent design:** a design in which the target object (the mentioned object) and the competitor object are not co-present on the same display. The target is absent when the competitor is presented. **Time bin:** an interest period is further divided into smaller ‘time bins’ (e.g., 50 ms bins), for which fixation proportion is computed or fixation is coded binomially. The binned fixation data are used for a time-course plot (e.g., Figs. 4, 6) and for testing fine-grained differences in the emergence of an effect over time (cf. section The temporal emergence of an effect).	
**Statistical terms** **Autocorrelation:** a tendency that fixation proportions between adjacent time bins are correlated with each other (cf. section Considerations in applying ANOVA /LME). **Categorical variable:** a variable which consists of categories (e.g., gender). **Coefficients:** the size of the coefficient of an independent variable indicates the effect that the independent variable has on the dependent variable. The sign of the coefficient indicates whether the independent variable has a positive or negative effect on the dependent variable. **Collinearity:** a situation where two independent variables are strongly correlated. When this applies to two or more variables, it is called ‘multicollinearity’ (cf. section Growth curve analysis (GCA)). **Degrees of freedom (*df*):** the number of observations that are free to vary when estimating a statistical parameter. For example, if three values have a mean of 5, and we are to determine each of these three values, then only two of them are free to vary (the first two can take on any value, but to obtain a mean of 5, the third number is fixed). The degrees of freedom in this case equals the number of values that are free to vary (two). **Dependent variable:** used synonymously with ‘outcome variable’. The variable that is measured and analysed. **Error:** a statistical error in a linear model means the difference between the observed value and the true value of an entire population parameter (a quantity which represents a property of the target population). Population parameters are usually impossible to know because an experiment usually does not recruit all members of a population. **False negative:** used synonymously with ‘type II error’. This occurs when a researcher believes that there is no effect of experimental condition when there is an effect. **False positive:** used synonymously with ‘type I error’. This occurs when a researcher believes that there is an effect of experimental condition when there is no effect. **Fixed effect:** an effect that is assumed to be relatively constant across individuals. **Independent variable:** used synonymously with ‘predictor variable’. The variable that is manipulated in an experiment. **Interaction effect:** combined effects of two or more independent variables on a dependent variable. **Intercept:** a constant corresponding to the value of the dependent variable when all independent variables are set to zero. **Interval variable:** a variable which requires that equal intervals on the scale represent equal differences in the property being measured. **Linear mixed-effects model (LME):** a linear model that involves estimating both fixed effects and random effects. This model considers the hierarchical structure of the data (where some variables are nested within other variables). **Main effect:** a unique effect of an independent variable on a dependent variable (ignoring effects of all other independent variables). **Ordinal variable:** a variable which consists of logically ordered categories (e.g., highest degree completed). **Parametric test:** a statistical test that requires parametric data. For data to be parametric, they must meet the following assumptions: (1) data are sampled from a normal distribution, (2) variances in each condition/group are approximately the same, or variances in the differences between conditions are approximately the same (for repeated-measures designs), (3) data should be measured at the interval or ratio level, (4) each observation is independent from the other (in between-group designs), or observations between different participants are independent (in repeated-measures designs; observations in experimental conditions within each participant can be non-independent), and (5) the dependent variable has an unbounded range. ANOVAs and *t*-tests are parametric tests. See section ANOVA, *t*-test for how to test these assumptions. **Permutation test:** a statistical test that involves obtaining the distribution under the null hypothesis (null hypothesis distribution) by resampling the data. The resampling is done by permuting (or ‘shuffling’) the data that are exchangeable under the null hypothesis. **Random effect:** an effect that is assumed to vary across individuals. **Ratio variable:** a variable which meets the requirement of an interval variable and additionally requires that the ratios of values alongside the scale be meaningful (e.g., reaction time). **Residual:** the difference between the observed value and the *estimate* of the true value. The true value is typically impossible to know but can be estimated from experimental data. A residual is closely related to the notion of an error, but they are different in that an error is a difference from the true value (not from its estimate). **Slope:** a slope indicates how much change in a dependent variable is associated with a one-unit increase in an independent variable. For example, a slope of .2 means that the dependent variable increases by .2 as one unit increases in the independent variable. **Sphericity:** a repeated-measures ANOVA assumes that the variances across conditions are equal and the covariances between pairs of conditions are equal (no two conditions are any more similar than any other two). This assumption is called the ‘assumption of sphericity’.	

#### Independent and dependent variables

Some VWP studies have employed a simple one-factor design, comparing eye movements to a target object between two or more so-called levels/conditions of the spoken stimuli. For example, Altmann and Kamide (1999) used one within-subject factor (verb selectional restrictions) with two levels/conditions (restrictive vs non-restrictive verbs). ‘Within-subject’ means that each subject (also called ‘participant’) is exposed to all the levels/conditions of a manipulated variable^4^. Tanenhaus et al. (1995) used a 2 × 2 within-subject design (two factors, each with two levels): structural ambiguity of the spoken sentences as a first factor (levels: ambiguous vs unambiguous) and the number of possible referents in the scene as a second factor (levels: one referent vs two referents). Other studies have included a between-subject variable, testing effects of socio-economic status on vocabulary growth (Fernald et al., 2013) or effects of age, or language proficiency on anticipatory eye movements (Gambi et al., 2018; Hopp, 2013; Ito et al., 2018b).

Typical dependent variables include fixations, inspections (or gaze), saccades and pupil size (cf. Glossary). Fixations refer to the period during which the eye gaze is stable on a single location and visual information is processed, which is presumably why a VWP analysis often focuses on fixation. Fixation data are often converted into proportion (called ‘fixation proportion’ or ‘fixation probability’) by computing the proportion of trials in which the fixation fell in an interest area (if a fixation falls in this area, it is treated as a fixation on the object corresponding to that interest area) (Dahan & Tanenhaus, 2004; Huettig & Altmann, 2005) or by computing the proportion of time spent fixating an interest area within a time window (which is then averaged across trials) (Borovsky et al., 2013; Chambers et al., 2004; Ito et al., 2018a). Inspections are computed by summing the duration of consecutive fixations to an interest area (Knoeferle & Crocker, 2006). Saccades refer to rapid, simultaneous movements of both eyes which occur between fixations. The proportion of trials in which a saccade was launched towards the target, or the saccade latency (the time taken to launch a saccade to the target interest area relative to a time point of interest; e.g., the onset of the target word) is sometimes analysed to explore attention shifts towards the target (Altmann & Kamide, 1999, 2004; Borovsky et al., 2012). Pupil size is often used as a measure of cognitive load, because it becomes larger (i.e., more dilated) as the spoken sentence becomes harder to process (Just & Carpenter, 1993). This measure has been applied to VWP studies, usually with adjustments to account for the gaze position (Carminati & Knoeferle, 2016; Demberg & Sayeed, 2016; Engelhardt et al., 2010; Scheepers & Crocker, 2004; Tromp et al., 2016).

In this paper, we focus on the analysis of fixations and inspections, but the analyses we present here can be applied to analysing pupil size or proportion of trials with saccades to the target (and their time-course). Fixation proportion is often used to visualise fixation bias towards one interest area over another. However, for parametric tests such as *t*-tests or ANOVA, using proportion data violates the assumption that the dependent variable has an unbounded range (because proportions are bounded from 0 to 1). It also violates the assumption that errors (cf. Glossary) are normally distributed and independent from the mean (because error variance is proportional to the mean) (Barr, 2008). Thus, using proportion data for an analysis can lead to improper estimation of effects (Jaeger, 2008). To deal with this issue, one can compute the empirical logit (a quasi-logit transformation) (Barr, 2008). The empirical logit has the advantages that it can filter out dependencies (autocorrelation) in eye-movement data (i.e., data at time X and data at time X+1 are highly correlated), and it excels in handling cases when the proportions are close to zero or 1 (see Donnelly & Verkuilen, 2017 for an alternative approach)^5^.

Alternatively, one can also code fixation binomially (1 = fixated, 0 = not fixated) for small time bins (cf. Glossary), and binomially-coded fixation can be used as a dependent variable in generalised linear-mixed models. The data are more likely to be binomially distributed when the interest period is short, as it is less likely that eyes move to another interest area during a short interest period (an interest period shorter than 100 ms may only contain one fixation, given what we know about the average duration of a fixation). In such a situation, it is often more suitable to use binomially-coded fixation. On the other hand, if the interest period is long, coding fixation binomially can produce very different results from those when proportion of time spent fixating on an interest area is computed. For example, if object A and object B were fixated for 600 ms and 200 ms, respectively, during an interest period of 1000 ms, the binomially-coded fixation would be 1 for both objects, whereas the fixation proportion would be .6 (= 600/1000) for object A and .2 (= 200/1000) for object B.

VWP studies often compare participants’ eye movements to one interest area over another. When two interest areas are co-present, comparing fixations to these interest areas with each other violates the assumption that data points for different conditions should be independent, because when one interest area is fixated, the other is not. A frequently used solution is to use log-ratio as a dependent variable (Arai et al., 2007). For example, to test fixation bias towards the target over the competitor, the log-ratio can be computed by the following formula: log((fixation proportion on target) / (fixation proportion on competitor)). Typically, a small value (e.g., .5 or .1) is added to both the numerator and the denominator to avoid failure of the computation when the denominator is zero. When the comparison is not between objects on the same scene, the fixations to these objects can be compared without computing a ratio. For example, when a target object is absent when a competitor is present and vice versa (i.e., target-absent design, cf. Glossary, section Visual stimuli), fixations to the target can be compared against fixations to the competitor.

#### Assignment of item–condition combinations to lists and counterbalancing

In a within-subject design, participants receive all conditions (levels). For example, in Altmann and Kamide (1999), there was one factor with two levels (restrictive condition vs non-restrictive condition). They created two lists using a Latin square, where participants received both restrictive and non-restrictive conditions, but not for the same item. In such a design, list 1 may contain items 1, 3, 5… in the restrictive condition, and items 2, 4, 6… in the non-restrictive condition; and list 2 may contain items 1, 3, 5… in the non-restrictive condition and items 2, 4, 6… in the restrictive condition (see Supplementary file S2 from https://osf.io/tzn8u/ for a template of a Latin square design). This allows researchers to avoid repeating a single item within a participant. Item repetition can allow participants to systematically track a pattern (e.g., when a scene appeared in the restrictive condition for the first time, it appeared in the non-restrictive condition for the second time), so repetition is often avoided (see Britt et al., 2014, for effects of repetition on anticipatory eye movements). However, repetition is sometimes inevitable when there are not many items (e.g., Gambi et al., 2018). When some items are repeated, it is desirable to ensure that the effects obtained are not due to repetition (e.g., by testing whether the effect was consistently found for the first and second/later instances of presentation of a stimulus). When there is an additional between-subject factor (e.g., age group), the stimuli are typically repeated across the participant groups.

Non-manipulated factors that could affect eye movements but are not of interest in a study are often counterbalanced. For example, participants might have a bias to look at the object on the left side over the object on the right side (for whatever reason). To ensure that the object position cannot explain biased looks, the object position is often counterbalanced (each object appears in each position equally frequently). Chambers and Cooke (2009) counterbalanced the gender of the object names so that the target and interlingual competitor shared gender in half of the items. Counterbalancing may also be done to the linguistic (auditory) stimuli. Knoeferle and Crocker (2006) used cross-spliced sentences in half of the items to exclude potential effects of intonational cues.

#### Visual stimuli

The visual stimuli typically consist of 2–5 objects. Experiments involving small children (about two years old or younger) tend to use a small number of objects (Fernald et al., 2013; Gambi et al., 2018; Mani & Huettig, 2012). Since the maximum number of objects that adults can efficiently process and actively remember is four on average (Cowan, 2001; Sperling, 1960), the inclusion of more objects in the visual scene is likely to interfere with language-mediated eye movements. For example, Ferreira et al. (2013; Experiment 1) replicated Tanenhaus et al. (1995) using four objects and found that participants hearing a locally ambiguous sentence (e.g., *Put the book on the chair in the bucket*) were more likely to look at the incorrect goal (e.g., chair) at the word *chair* when there was only one book than when there were two books (though the effect does not seem consistent in the time-course graph). However, when the visual scene contained eight additional (12 total) objects (Experiment 4), the effect did not replicate and showed (numerically) an opposite pattern (see also Hintz & Huettig, 2015, for effects of visual complexity on phonological, semantic and shape competitor effects).

Many of the VWP studies used scenes containing line drawings of objects arranged in an array. These scenes are different from natural scenes in several ways including scene complexity, the quality of the image and the size of the objects. An important consideration is that object identification is much quicker in a visual array with 4–5 objects than in natural scenes with many more objects (although people can derive the gist, or semantically interpret a natural scene within 30–50 ms) (Henderson & Ferreira, 2004). As people use linguistic and visual information in combination in a VWP setting (e.g., Knoeferle et al., 2005), linguistic processing is subject to the influence of visual information if participants have identified some or all of the depicted objects before hearing the linguistic information. Thus, the influence of the visual information must be considered when drawing conclusions from a VWP study.

The visual stimuli can be printed words instead of objects. Studies on phonological and orthographic processing used the printed-word VWP and replicated phonological, orthographic and semantic competitor effects found in the standard VWP (Ito, 2019; McQueen & Viebahn, 2007; Salverda & Tanenhaus, 2010; Shen et al., 2016; Veivo et al., 2016). The use of printed words allows researchers to use abstract words that are hard to depict as visual stimuli. When researchers are interested in effects that hinge on a high naming agreement (e.g., phonological competitor effect), the printed-word VWP may be considered as a better alternative. However, researchers must be aware that the type of visual stimuli can affect language-mediated eye movements. For example, phonological competitor effects tend to be more robust in the printed-word VWP (but see Apfelbaum et al., 2021), whereas semantic or shape competitor effects tend to be more robust in the standard (depicted-objects) VWP (Huettig & McQueen, 2007).

In studies testing a competitor effect, the target and the competitor may be co-present in the visual scene, or the target may be absent when the competitor is present (*target-absent design*, cf. Glossary). In studies testing disambiguation (Knoeferle et al., 2005; Tanenhaus et al., 1995) or how activation of different types of information competes with each other (Allopenna et al., 1998; Huettig & McQueen, 2007), all critical objects (e.g., the target and competitor objects, potential referents) are often presented in the same scene. The target-absent design has an advantage in preventing fixations on the competitor being swamped by fixations on the target (Huettig & Altmann, 2005), so it is suitable when the effect of interest may be subtle (e.g., Rommers et al., 2013). However, a downside of this design is that there may be more noise in the data due to between-trial comparisons, and statistical power may be reduced relative to within-trial comparisons. The decision about this design affects the choice of the dependent variable (as discussed in section Independent and dependent variables). The fixations to the target and the competitor can be directly compared against each other in a target-absent design (because fixations are independent) and indirectly via log-ratios if the target and the competitor are co-present (because fixations are non-independent).

#### Preview and speech rate

The preview time (how long the visual scene is shown relative to the occurrence of a critical spoken input) and speech rate can affect eye movements considerably. It is common to give participants some time to preview the scene before the auditory stimulus is presented because people tend to react more slowly to (i.e., need more time to process) visual than auditory stimuli (Teichner, 1954). In addition, eye movements upon the presentation of the scene are largely driven by visual features of the objects presented (Henderson & Ferreira, 2004). If there is a preview time, these eye movements are more likely to occur during the preview and are less likely to interfere with eye movements during the presentation of the auditory stimulus. Dahan and Tanenhaus (2005) tested whether the preview time affects a shape competitor effect using a 300 ms or 1000 ms preview. They found a shape competitor effect in both preview conditions, but the effect was larger in the longer preview condition. Huettig and McQueen (2007) investigated how the preview time interacts with phonological, semantic and shape competitor effects. They found phonological, semantic and shape competitor effects when the visual scene was presented at the sentence onset (the target word appeared after seven words on average), but there were only semantic and shape competitor effects when it was presented 200 ms before the target word onset (Experiments 1–2). Apfelbaum et al. (2021) investigated which processes were affected by varied preview and demonstrated the benefit of preview as it can prevent early eye movements, driven by processes that may not be of interest (e.g., visual search, strategies) from adding noise to eye movements driven by processes of interest (e.g., linguistic processes like word recognition).

It is also common to use a relatively slow speech rate, as language-mediated eye movements tend to be reduced when the speech is fast (vs slow). Huettig and Guerra (2019) investigated the effects of preview time and speech rate on anticipatory eye movements. Native Dutch speakers listened to Dutch sentences such as *Kijk naar de*_-common_ *afgebeelde fiets*_-common_ (‘Look at the_-common_ displayed bicycle_-common_’) while viewing a scene containing the target object (a bicycle) and three distractor objects. The speech rate was manipulated within items, and the same set of sentences were presented at either a normal speech rate (1.8 seconds/sentence on average) or a slower rate (4.2 seconds/sentence on average). The target had a common gender (hence required an article *de*) and the distractors all had a neuter gender (hence required an article *het*) or vice versa, so the target object was predictable based on the gender of the article. Participants looked at the target object in both speech rate conditions before it was mentioned when the scene was presented four seconds before the sentence onset (Experiment 1), suggesting that they anticipated the target based on the gender-marked article. However, when participants had a shorter, one-second preview, this effect was found only at a slow speech rate (Experiment 2).

Thus, it is important to give participants enough time to preview the scene before the target word is presented. However, in some studies the scene appeared shortly before the target word onset (i.e., .5–1-second preview) in order to minimise priming from the visual scene. Dahan et al. (2001) used a 500 ms preview to make it less likely that participants would name the depicted objects implicitly (note that a recent study suggests participants are unlikely to do so during a preview; Apfelbaum et al., 2021). Other studies investigating pre-activation of certain information from the sentence context (Ito et al., 2018b; Rommers et al., 2013) used a short preview time to make it less likely that the pre-activation arose from the preview. If researchers are to adapt this presentation, it is important to ensure that there is no baseline bias (meaning that participants fixate one object more than another already before a critical time window); the existence of a baseline bias must be verified because eye movements immediately after the presentation of the visual scene can contain fixation bias due to visual features (cf. Barr et al., 2011). In studies on prediction based on a linguistic cue, it is also important to ensure enough time between the prediction-cueing word (e.g., a gender-marked article in Huettig & Guerra, 2019) and the target word. Many studies inserted additional semantically non-constraining words (e.g., Hopp, 2013; Huettig & Guerra, 2019; Kamide et al., 2003) to extend the time window during which looks to the target can be interpreted as indicating anticipatory processes.

#### Time window

The interest periods (time windows for the analysis, cf. Glossary) should be defined before data collection (and ideally preregistered) irrespective of which analysis is used, because the choice of an interest period can affect statistical results (Peelle & Van Engen, 2021). Studies on word-level processing often take the time window from the acoustic onset to the offset of the target word, or to 1–2 seconds after the target word onset if the target word duration is relatively short. If the word duration is relatively short and if the interest period is cut off at the word offset, an effect of interest may not be evident or may not reach a peak before the target word offset (e.g., Allopenna et al., 1998); to capture delayed effects, a longer time window can be used for the analysis. Eye movements as a response to a target word are often observable within one second from the target word onset (Allopenna et al., 1998; Yee & Sedivy, 2006). However, when the effect is expected to be delayed (e.g., when testing less proficient language speakers), the interest period may be extended to capture a delayed effect (Mirman et al., 2011; Yee et al., 2008). Since it takes around 200 ms to launch a saccade in response to a stimulus (Saslow, 1967), the time window is sometimes shifted 200 ms forward (e.g., from 200 ms after the word onset to 200 ms after the word offset) (but see Altmann, 2011, for a shorter estimate).

Studies on disambiguation may take the window from the onset of a temporarily ambiguous region (e.g., *on the towel* in *Put the apple on the towel in the box*) to the onset of a disambiguating region (e.g., *in the box*) to test an initial interpretation of the ambiguous phrase (Tanenhaus et al., 1995) or take a region where disambiguation is possible (e.g., after the verb *wäscht* in *Die Prinzessin*_-Subj/Obj_ *wäscht offensichtlich den Pirat*_-Obj_; ‘The princess_-Subj/Obj_ is apparently washing the pirate_-Obj_’, for the scene where a princess is washing a pirate) to test whether people attempt a disambiguation before the thematic role relations are fully disambiguated by case marking on the post-verbal noun phrase (Knoeferle et al., 2005). Studies on anticipatory processing have taken an interest period from the onset of a word that makes an upcoming object predictable (e.g., *eat* in *The boy will eat the cake* for the scene where the cake is the only edible object) to the onset of the predictable word (e.g., *cake*). They may also take a short window before the onset of the predictable word (Ito et al., 2018b; Rommers et al., 2013) or the entire sentence context window up to the onset of the predictable word (Kukona, 2020) if the word is predictable from the sentence context.

### Descriptions of our data sets

In addition to considering the aspects discussed in section Design, visual and speech stimuli, timing, interest periods and task, key decisions must also be made in analysing VWP data. Below we present two example data sets (sections Ito et al. (2018b) and Knoeferle and Crocker (2006; Experiment 1)), utilising the experimental terminology and characteristics introduced in section Design, visual and speech stimuli, timing, interest periods and task; for these, we illustrate eye-movement data analysis in sections Analysis of fixation averages for individual interest periods (for interest period averages) and The temporal emergence of an effect (for time course).

#### Ito et al. (2018b)

The first data set is from Ito et al. (2018b). They investigated prediction of a specific word based on a sentence context in first-language (L1) and second-language (L2) English speakers (with L1 Japanese). The auditory stimuli were sentences containing a highly predictable word (e.g., *cloud* in *The tourists expected rain when the sun went behind the cloud,* …). The visual stimuli contained four objects, one of which was the critical (i.e., manipulated, non-distractor) object. The independent variable was the critical object condition (four levels: target, English competitor, Japanese competitor and unrelated). The critical object represented the target word (target condition, e.g., *cloud*), an English competitor which shared initial phonemes with the target word (English competitor condition, e.g., *clown*), a Japanese competitor whose name shared initial mora(s) with the target word when translated into Japanese (Japanese competitor condition, e.g., competitor: *bear*; Japanese: ‘kuma’; target: *cloud*; Japanese: ‘kumo’) or an unrelated word (unrelated condition, e.g., *globe*). The English competitor was included to test pre-activation of phonology. The participant group (two levels, L1 and L2) was a between-subject independent variable. The dependent variable was fixation proportions to the critical object (or binomially-coded fixation, see the analysis sections Analysis of fixation averages for individual interest periods and The temporal emergence of an effect for how the dependent variable was computed for each analysis).

This study used a target-absent design. Participants only saw one of the critical objects (together with the same three distractors) for each item. Thus, fixation proportions to critical objects can be compared against each other without computing a log-ratio. Four experimental lists were constructed following a Latin square design. Participants received only one condition per item and received the same number of trials for each condition. The objects appeared on the screen 1000 ms before the target word onset. No pre-sentence preview was used to minimise a potential effect of phonological priming from the object names. The sentences were spoken slowly with phrases spaced out in time, at a rate of approximately 2.6 syllables per second. To investigate prediction (processes happening before the occurrence of the predictable target word), a time window before that word was used for the analysis (see sections Analysis of fixation averages for individual interest periods and The temporal emergence of an effect for a specific time window used in each analysis).

If participants activated the phonological information of the target word before they heard it, the English competitor was expected to attract more looks than the unrelated object (due to a phonological competitor effect; cf. Allopenna et al., 1998). The Japanese competitor was included to test interlingual competition (cf. Spivey & Marian, 1999). If L1 Japanese-L2 English participants pre-activated the phonological information of the target word in Japanese, the Japanese competitor was expected to attract more looks than the unrelated object (e.g., a globe).

The results showed that L1 English speakers were more likely to look at the target object and the English competitor object over the unrelated object before the target word was mentioned, suggesting that they predicted the phonological information of the target. L1 Japanese speakers also showed biased looks to the target and the English competitor, but their looks were delayed compared with L1 English speakers, and the English competitor effect occurred well after the target word had been mentioned. The Japanese competitor was equally likely to be fixated as the unrelated object in both participant groups. These findings suggest that L1 Japanese speakers predicted some information (e.g., meaning) about the target word (albeit slightly later than L1 English speakers), but there was no evidence that they predicted phonological information of the target word in English or Japanese.

#### Knoeferle and Crocker (2006; Experiment 1)

The second data set we use is from Knoeferle and Crocker (2006; Experiment 1), whose study was based on Knoeferle et al. (2005). Knoeferle and Crocker (2006; Experiment 1) tested the effects of depicted actions and events on incremental thematic role assignment and replicated the findings in Knoeferle et al. (2005) using English main clause vs reduced relative ambiguity. They used sentences such as *The ballerina splashed apparently the cellist*… (main clause condition) and *The ballerina splashed apparently by the cellist*… (reduced relative condition) in combination with a scene depicting a ballerina either splashing or being splashed, respectively. The independent variable was the sentence condition (two levels: main clause and reduced relative), and the dependent variable we used for the present analysis was a log-ratio of inspections to the agent vs patient in this study (looks to the agent and patient were not independent). A semantically non-constraining adverb extended the time window during which looks to the agent/patient could be interpreted as indicating anticipatory thematic role assignment.

The visual scenes were analogous to those in Knoeferle et al. (2005) and depicted atypical action events (e.g., a princess splashing a cellist and being sketched by a fencer, or a princess being splashed by a cellist and sketching a fencer). The above two versions of the visual scene (and their mirrored versions, as well as a counterbalancing scene in which agent/patient role-depiction of characters was reversed) and corresponding sentences were created for each item to counterbalance the position of the characters and their role.

To further counterbalance the intonational cues, half of the critical sentences were cross-spliced up to and including the adverb. The scene appeared on the screen 1000 ms before the sentence onset (= 1000 ms preview time). Knoeferle and Crocker (2006; Experiment 1) analysed three time windows: the verb window (from the verb onset to the adverb onset), the adverb window (from the adverb onset to the onset of the second argument) and the second argument window (the second noun phrase in the main clause condition and the prepositional phrase in the reduced relative condition) (see sections Analysis of fixation averages for individual interest periods and The temporal emergence of an effect for a specific time window used in each analysis).

As in Knoeferle et al. (2005), an early disambiguation of the thematic role (after the verb) was only possible if listeners integrated both the linguistic and visual information. Upon hearing the verb, participants were more likely to look at the patient of the verb, suggesting a general preference for the main clause interpretation. At the adverb, participants were more likely to look at the patient in the main clause (vs reduced relative) condition and the agent in the reduced relative (vs main clause) condition. The following noun region showed the same interaction. The effect at the adverb demonstrates that participants disambiguated the structure quickly before the information that allowed structural disambiguation (the grammatical object or the *by*-phrase) became available.

## Analysis of fixation averages for individual interest periods

In this section, we present two examples of analysing fixation data in a predefined time window. Detailed steps and R codes are available at the Open Science Framework (https://osf.io/tzn8u/). The README file there also contains some basic R tips.

### Data preparation

When preparing recorded eye-movement data for analysis, the researcher must choose how to treat (very short and very long) fixations and blinks. Current software (e.g., SR Research Data Viewer) contains filtering options that facilitate such preprocessing. Interested readers can refer to YouTube videos from SR Research (https://youtu.be/pM_dxz-G_ic). There are also several R packages that are helpful for data preparation, such as gazeR (Geller et al., 2020), eyetrackingR (Dink & Ferguson, 2015) and VWPre (Porretta et al., 2020).

In Ito et al. (2018b), blinks and fixations out of the interest areas were coded as 0 (i.e., no fixation on any interest area) and included in the data. In Knoeferle and Crocker (2006), consecutive fixations within one interest area were merged and counted as one *inspection*. Consecutive fixations of less than 80 ms were also merged, and blinks and fixations out of the interest area were added to the immediately preceding fixations. These are common preprocessing steps, as short fixations may follow an erroneous saccade (Findlay & Brown, 2006), and blinks can interrupt a fixation (one fixation that contains a blink will appear as two separate fixations in the recorded data). In other studies, blinks and fixations out of the interest areas were excluded from the analysis (e.g., Bosker et al., 2014; Silva et al., 2013). This variability in the previous studies suggests a potential need for more explicit homogenisation of the preprocessing steps.

### ANOVA, *t*-test

**Fig. 1:**
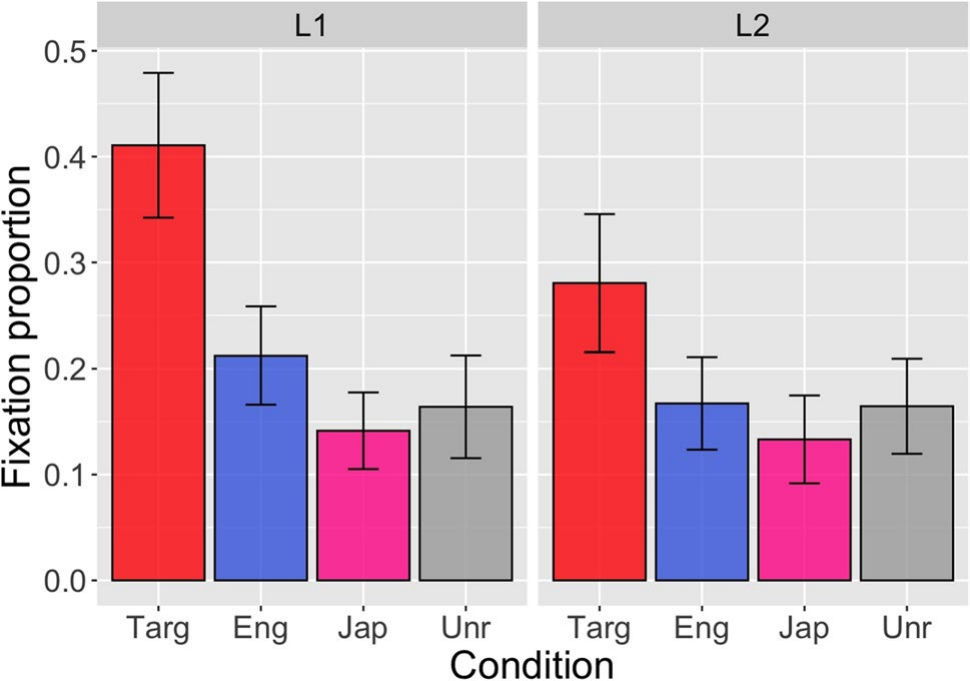
Mean fixation proportions for each condition (Targ = target, Eng = English competitor, Jap = Japanese competitor, Unr = unrelated) and group (L1 vs L2) in the interest period from −800 ms to 0 ms relative to the target word onset. The data are from Ito et al. (2018b). The error bars represent standard errors.

In this tutorial (IPC_ANOVA.html, IPC_ANOVA.Rmd), we use the eye sample data from Ito et al. (2018b) (see section ﻿Ito et al. (2018b) for details), exported using the SR Research Data Viewer. The data file (IPC_fix_-800_0.txt) contains the sample counts on each interest area, the total number of blink samples and off-screen samples, and the proportion of samples on critical interest areas as well as the experimental variables including subject ID, item ID, trial number, condition and language group. It contains data in the interest period from 200 ms after the onset of the visual scene (800 ms before the target word) to the onset of the target word. Figure 1 plots the mean fixation proportions (proportion of time spent fixating on the critical object) for each condition and group in this time window.

#### Verifying assumptions

ANOVA or *t*-tests are parametric tests which require the data to meet certain assumptions. One of the assumptions is that the dependent variable be measured at least at the interval level (cf. Glossary). The fixation data are categorical (an object is either fixated or not at a particular time point), and many articles have reported transforming categorical fixation data to continuous proportion data. The proportion data, however, violate the assumption that the dependent variable has an unbounded range (because proportions are bounded from 0 to 1). To resolve this issue, we transformed the fixation data to a continuous variable by computing the *empirical logit* (Barr, 2008) (see section Independent and dependent variables for discussion on dependent variables). The formula for computing the empirical logit is log((Y+.5)/(N-Y+.5)), where Y is the total number of samples that fell in the critical interest area, and N is the total number of samples for the interest period.

Another assumption of parametric tests is that the variances in each condition/group are the same (homogeneity of variance). This can be tested using Levene’s test for equality of variances (Field et al., 2012). For the 4 (condition) × 2 (group) design in this study, we tested whether the variances did not differ between groups at each level of condition. This test showed a significant result for the target condition, *p* = .005, so the homogeneity of variance assumption is not tenable.

We then tested the normal distribution assumption—whether the sampling distribution of the means for each condition within each group was normal (for significance tests to be accurate) (Tabachnick & Fidell, 2007) and whether the distribution of the residuals was normal (for the estimates of the parameters in the model to be optimal) (Field et al., 2012) using the Shapiro–Wilk test. The tests showed that the distribution of the means was significantly different from the normal distribution in all conditions in both groups, *p*s < .05, and the distribution of the residuals was marginally significantly different from the normal distribution, *p* = .059. This is not surprising because transforming the data using empirical logit does not make the data normally distributed. We included this test only for didactic purposes.

Since the data do not meet the homogeneity of variance assumption and the normal distribution assumption, an alternative test should be used. Transforming the data can be helpful when the data are skewed, but when the data are categorical, using ANOVA could lead to spurious results (Jaeger, 2008). We present ANOVA and *t*-test below only for demonstration purposes. In Ito et al. (2018b), growth curve analysis was used (cf. section Growth curve analysis (GCA)). When the assumption of sphericity (cf. Glossary) was violated, we used the Greenhouse–Geisser correction.

#### Code for the ANOVA analysis and *t*-tests

The data need to be aggregated over subjects (for a by-subject analysis) or over items (for a by-item analysis) before they are entered into ANOVA or *t*-tests. We ran a mixed ANOVA testing main effects of condition and group as well as their interaction using the *aov_ez* function from the *afex* package (Singmann et al., 2021) using the code below (elogFix = empirical-logit transformed fixation proportion, Lang = Language group).

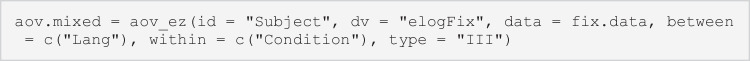



The effect of condition was significant, *F*(2.5, 114.83) = 18.8, *MSE* = 2.7, *p* < .001. There was no significant effect of group, *F*(1, 46) = 3.3, *MSE* = 5.8, *p* = .08, or interaction of condition by group, *p* = .2.

Next, we ran paired *t*-tests as follow-up analyses. Strictly speaking, these tests should be conducted to resolve which of the comparisons between individual levels/conditions in a significant interaction are reliable. There was no significant interaction in the current analysis, and in that situation no further tests need to be conducted, but we present follow-up tests for demonstration purposes. An example code (for a by-subject analysis comparing the target vs unrelated conditions in the L1 group) is below:

 In the L1 group, the target object was more likely to be fixated than the unrelated object, *t*(23) = 4.6, *p* < .001 (by-subject analysis), *t*(13) = 6.4, *p* < .001 (by-item analysis). The differences between the English competitor and the unrelated conditions and between the Japanese competitor and the unrelated conditions were not significant, *p*s > .09. The null effect for the English competitor vs unrelated condition was different from the results of the growth curve analysis (cf. section Growth curve analysis (GCA)), which showed that the English competitor object was more likely to be fixated than the unrelated object. In the L2 group, the target object was more likely to be fixated than the unrelated object, *t*(23) = 2.8, *p* = .01 (by-subject analysis), *t*(15) = 2.3, *p* = .03 (by-item analysis). Similar to the L1 group, the differences between the English competitor and the unrelated conditions and between the Japanese competitor and the unrelated conditions were not significant, *p*s > .3. These results suggest that L1 and L2 speakers pre-activated some representations of the target word before it was mentioned, but there was no evidence that they pre-activated phonological information.

### LME

**Fig. 2:**
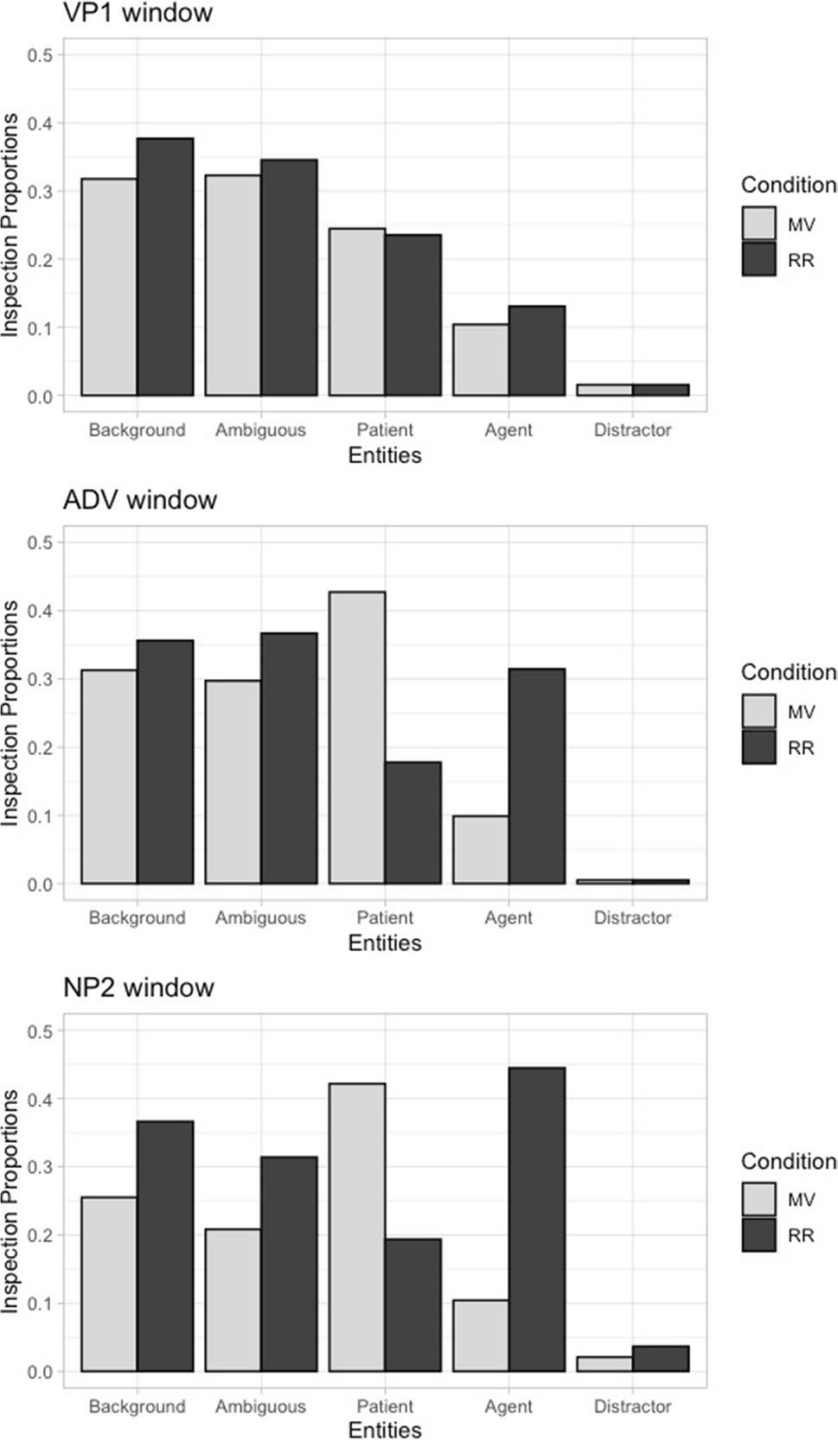
Mean inspection proportions to each entity for each condition (MV, RR) and interest period (VP1, ADV, NP2). The data are from Knoeferle and Crocker (2006)

In the LME tutorial (KC_LME.html, KC_LME.Rmd), we use the data from Knoeferle and Crocker (2006; Experiment 1a). Their study used temporarily ambiguous sentences (e.g., *The ballerina splashed apparently the cellist/by the cellist*…, the verb can be a main verb [MV condition] or a reduced relative [RR condition]) and tested whether people use visual information together with linguistic information to resolve the local syntactic ambiguity before hearing the second noun (see section Knoeferle and Crocker (2006; Experiment 1) for details). The scene contained a role-ambiguous character (a ballerina) and possible agent and patient (a cellist, a fencer). The data file (KC_window_binomial.txt) contains the binomially-coded inspection data (1 = inspected, 0 = not inspected) for each role character in each interest period. The interest periods we analyse here are the VP1 (the ambiguous verb, *splashed*), the ADV (the adverb, *apparently*) and the NP2 (the disambiguation phrase, *the cellist*/*by the cellist*). The details about the interest periods and counterbalancing can be found in the tutorial file (KC_LME.html). Figure 2 plots the mean proportions of inspections (proportion of trials in which there was an inspection on the interest area).

The LME can model by-subject and by-item variability simultaneously by including random intercepts (to model variability across subjects or items) and random slopes (to model variability in the size of an effect across subjects or items), so the variances can be allowed to vary across conditions or groups. In this tutorial, our models only included by-subject and by-item random intercepts for the sake of simplicity (i.e., only the variances across subjects and items were allowed to vary). An alternative would be to start with a fully specified model (random intercepts and random slopes for all main effects of the independent variables and their interaction to capture the associated variability by subjects and by items), assuming a hypothesis-testing approach (Barr et al., 2013). The best model structure (which random effects are included) should be determined based on the study design (cf. section Assignment of item-condition combinations to lists and counterbalancing) and model fit (for guidance on how to choose a random-effects structure and how to deal with singular fits, see Barr, 2013; Barr et al., 2013; Bates et al., 2015; DeBruine & Barr, 2021; Matuschek et al., 2017). For example, a random slope for condition by subject would capture the variability in the effect of condition by subject. Thus, researchers may want to include it if the study design allows such variability (i.e., some subjects might be expected to show a larger effect of condition than others). On the other hand, including a random slope for condition by subject would not be appropriate if condition were a between-subject factor (i.e., each subject contributes to only one out of the two (or more) conditions). In the latter case, it would be impossible to measure an effect of condition within each subject.

For the dependent variable, we computed a log-ratio of inspection proportions to the agent vs patient using the formula log((Agent inspection+.5)/(Patient inspection+.5)) to quantify a fixation bias towards the agent vs patient. A positive value indicates that there were more inspections to the agent than the patient, and a negative value indicates that there were more inspections to the patient than the agent. The value zero indicates that there was no fixation bias. We tested whether the log-ratio was significantly different from zero (to test whether there was fixation bias) and whether it was significantly different between the MV and RR conditions. The categorical variable condition was sum-coded to compare the two conditions against each other (see Schad et al., 2020, for a tutorial on contrast coding in R). The example codes for both the former test (for the MV condition) and the test for the effect of condition in the verb window are given below (log_AP = agent vs patient log-ratio):

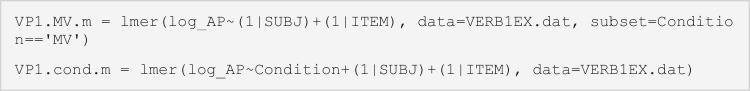



If we wanted to start from the model with a maximal random-effects structure (for the model testing the effect of condition), the R syntax would be the following:

 In the VP1, the model for each condition showed that the intercept was significantly different from zero in the MV condition, *β* = −.15, *SE* = .049, *t* = −3.1, and the RR condition, *β* = −.11, *SE* = .050, *t* = −2.3. The mean log-ratio was negative in both conditions, suggesting that there were significantly more inspections to the patient than the agent. The model testing the effect of condition did not show a significant effect of condition, *β* = −.02, *SE* = .032, *t* = −.6, suggesting that the fixation bias towards the patient was similar in the MV and RR conditions. In the ADV, the model for each condition also showed that the intercept was significantly different from zero, *β* = −.36, *SE* = .062, *t* = −5.8 (MV condition), *β* = .15, *SE* = .060, *t* = 2.5 (RR condition). The log-ratio was negative in the MV condition and positive in the RR condition, suggesting that there were more inspections to the patient in the MV condition but to the agent in the RR condition. This pattern was statistically supported by a significant effect of condition, *β* = −.25, *SE* = .036, *t* = −7.1. The NP2 showed results consistent with the ADV. The model for each condition showed that the intercept was significantly different from zero, *β* = −.35, *SE* = .052, *t* = −6.7 (MV condition), *β* = .28, *SE* = .055, *t* = 5.0 (RR condition), and the log-ratio was negative in the MV condition and positive in the RR condition. The effect of condition was significant, *β* = −.31, *SE* = .037, *t* = −8.5. Thus, the fixation bias to the patient in the MV condition and to the agent in the RR condition in the ADV continued in the NP2. These results replicate the results from hierarchical log-linear models reported in Knoeferle and Crocker (2006). The similar bias towards the patient in both MV and RR conditions during the VP1 arguably suggests listeners’ preference for the subject–verb–object (SVO) (vs object–verb–subject [OVS]) interpretation. The interaction in the ADV suggests that listeners used both linguistic and visual information to resolve the local syntactic ambiguity before the disambiguation was possible based purely on the linguistic information (NP2).

## The temporal emergence of an effect

In section Analysis of fixation averages for individual interest periods, we described methods for analysing differences in averaged fixation proportion or log-ratio of inspections in a predefined interest period. These analyses do not reveal whether the differences changed over time within an interest period, or precisely when the differences started to emerge. The temporal emergence of an effect is often critical for psycholinguistic research, as psycholinguistic models aim at accommodating processes that occur within a few hundred milliseconds as a word (or / in a sentence) unfolds, which can be difficult to detect using other behavioural measures such as linguistic judgement/rating or reaction times. Researchers may want to test whether the effect occurs earlier in one group compared with another, or whether the effect occurs prior to the onset of a critical word or within an interest period. The VWP is well suited to test such hypotheses, since currently available eye-trackers can track people’s eye movements at every millisecond, allowing researchers to obtain very fine-grained time-course data. However, as we see below, testing fine-grained, emergent time-course differences using the ANOVA or LME is not straightforward. In this section, we first discuss the nature of the VWP data we consider for analysing the temporal emergence of an effect, and why conducting an ANOVA or LME is often not the best choice for that analysis.

### Considerations in applying ANOVA/LME

One important characteristic of the VWP data is autocorrelation (cf. Glossary). While an eye-tracker can record eye -movements at every millisecond, people do not move their eyes every millisecond (fixations typically last 200–300 ms). Thus, data in adjacent time points tend to be correlated. For example, Stone et al. (2020) demonstrated autocorrelation using their data and showed that the correlation coefficient for two adjacent bins was over .9, over .8 and over .75 when the data were binned for every 50 ms, 100 ms and 150 ms, respectively. Thus, even if the observations were grouped into relatively large time bins (e.g., 150 ms), the autocorrelation was not completely eliminated.

In the analyses we conducted in section Analysis of fixation averages for individual interest periods, we aggregated the data for the selected interest periods, but by doing so we lost its finer-grained temporal resolution. An intuitive approach might be to apply an ANOVA or LME in multiple predefined interest periods or include interest period as a predictor. However, this approach ignores the autocorrelation, violates the assumption of independence of the observations (assumed in parametric tests) and can inflate the type I error (false positive) rate (Huang & Snedeker, 2020). A type I error refers to thinking there is an effect when there is none (cf. Glossary). Moreover, if the analysis is conducted in many small interest periods (e.g., every 50 ms), it can increase the type I error rate due to multiple analyses being conducted (every time an analysis is conducted, the likelihood of a false positive is < 1 − .95 = .05, but with every analysis added, this likelihood changes, e.g., for three analyses, 1 − (.95*.95*.95) = 1−.857 = .143; after three analyses, the likelihood of a false positive is no longer < .05, but only < .14). The multiple comparisons problem can be corrected (e.g., Bonferroni correction), but the correction can be too conservative and may increase the type II error rate (cf. Glossary).

An alternative approach may be to use each word in sentences as individual interest periods and run an ANOVA or LME over these time interest periods, but this approach still involves multiple comparisons. A similar approach that is less problematic would be to run the analysis in only a few interest periods in which an effect of interest is expected to occur, as the type I error (if no correction is applied) or type II error (if a correction is applied) would increase to a lesser extent. While this approach can test whether the effect is different between/across these interest periods, testing finer-grained differences in the emergence of an effect over time is not straightforward. If researchers select the interest period for an analysis after data collection, they could arbitrarily take one where the effect is likely to be significant (i.e., the researcher degrees of freedom problem) (cf. section Time window). This can increase the possibility of a type I error and the results may not replicate (Simmons et al., 2011). In sum, we would want to control for autocorrelation of the time-course data and avoid inflated type I (and type II) error rates associated with multiple tests across time (and corrections for these).

**Fig. 3:**
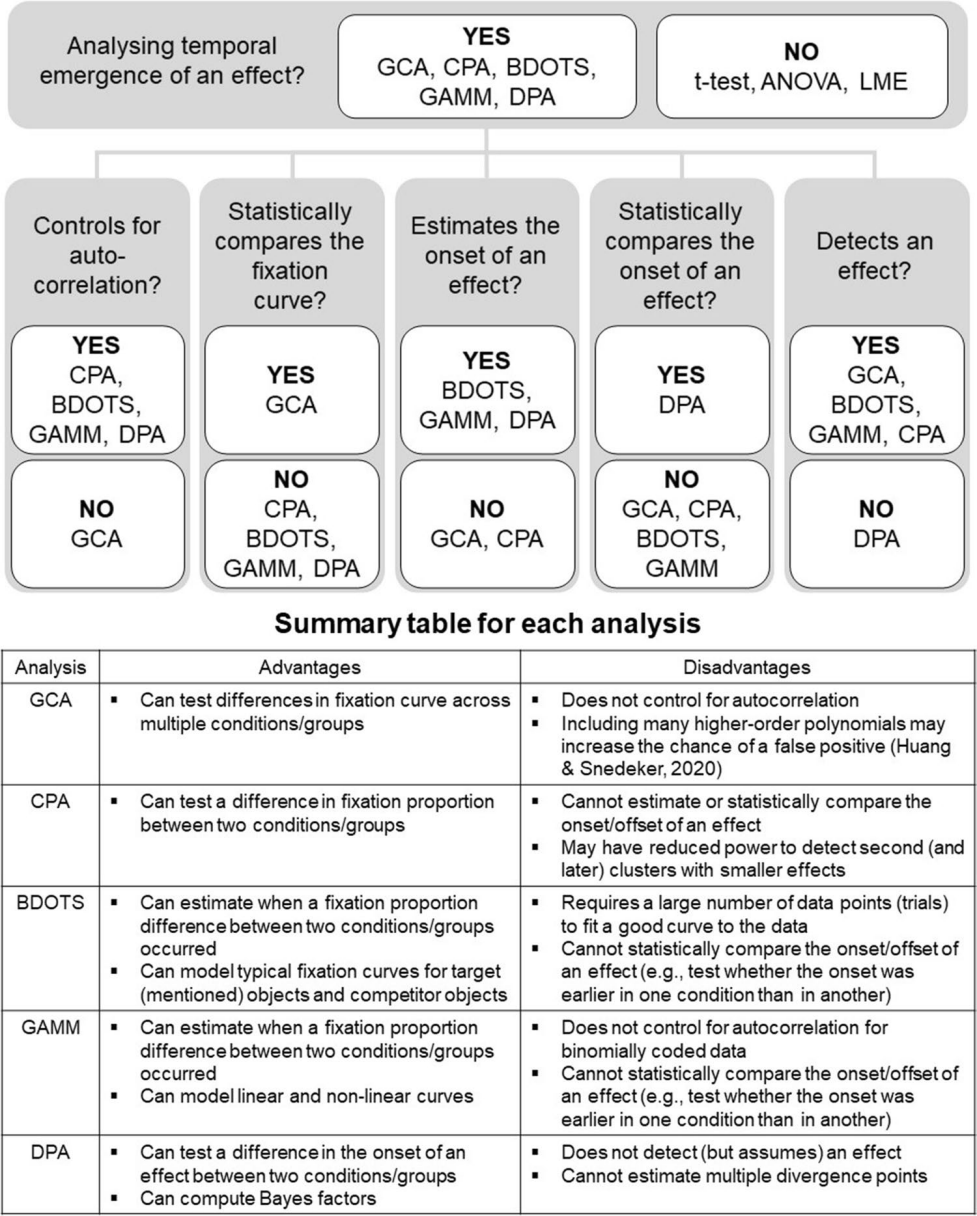
Overview of the VWP analysis methods. LME: linear mixed-effects model, GCA: growth curve analysis, CPA: cluster-based permutation analysis, BDOTS: bootstrapped difference of timeseries, GAMM: generalised additive modelling, DPA: divergence point analysis

In the following sections, we compare five alternative approaches to analysing the temporal emergence of an effect: the growth curve analysis (GCA) (Mirman et al., 2008), the cluster-based permutation analysis (CPA) (Maris & Oostenveld, 2007), the bootstrapped differences of timeseries (BDOTS), the generalised additive modelling (GAMM) and the divergence point analysis (DPA) (Stone et al., 2020). We will describe the advantages and disadvantages of each method and demonstrate the analysis steps for the example data sets we used in section Analysis of fixation averages for individual interest periods. Figure 3 shows a summary of the characteristics of these analyses.

The GCA is the only method among the three that can model how an effect changes over time, so it is recommended for studies that have hypotheses about the dynamics of an effect emerging over time (e.g., from word onset to word offset). However, this method does not control for autocorrelation of eye-movement data (Huang & Snedeker, 2020). Eye-movement responses obtained across small time bins (used in the GCA) are arguably highly correlated such that autocorrelation of the data points is particularly challenging when examining the emergence of effects within words. To test for the emergence of an effect within a word, methods that control for autocorrelation would be particularly suitable. The CPA and the DPA control for autocorrelation, as the temporal structure of the data is respected during the permutation or resampling. The BDOTS controls for autocorrelation by estimating the autocorrelation between test statistics and using it to adjust the α value. The GAMM also estimates the autocorrelation and controls for it by including it in the model.

The CPA, the BDOTS and the GAMM can detect the statistical significance of an effect (whether there is a statistically significant difference in the proportion of fixations/inspections between conditions/groups), while the DPA cannot. The BDOTS, the GAMM, and the DPA can estimate the onset of an effect, while the CPA cannot. The BDOTS and the GAMM can estimate in which time bins an effect was significant, so they are suitable if researchers are interested in how long an effect lasted. The DPA can test whether the onset of an effect is significantly different between two conditions or groups, while the BDOTS and the GAMM cannot. These methods can be used complementarily to test different questions. For example, researchers interested in whether there is an effect of condition and when the effect started to emerge could run a CPA to determine whether there is a significant effect and then run a DPA to determine or compare its onset. However, using one analysis to select a time window and then running a second analysis testing the same question on the selected time window would be double-dipping and should be avoided (Kriegeskorte et al., 2009).

### Growth curve analysis (GCA)

The GCA can analyse changes in fixation proportion over time (Mirman et al., 2008). 

#### Advantages of GCA

It is particularly suitable for analysing dynamic changes in the emergence of an effect in eye movements. Assume that ‘an effect’ here refers to changes in looks to one compared with another object. For example, researchers may want to test whether the fixation proportion to one object increases more quickly than that to another object, or whether the fixation proportion to one object reaches a peak (i.e., increases and then decreases) earlier than that to another object.

Purely linear models (like ANOVA or LME) cannot capture changes from increase to decrease (or vice versa) in fixation proportion —they model eye movements as data points along a single line with a fixed slope. To go beyond such a linear model, the GCA captures eye-movement changes (e.g., an increase followed by a decrease in looks to an object). It achieves this by using so-called power polynomials; these can represent not just a linear but also a curvilinear relationship (fixation proportion with one or more peaks) between fixation proportion and time. Power polynomials can be computed by raising the variable ‘time’ to a particular power. For example, a quadratic curve (a curve with a single inflection) can be represented by including time^2^, and a cubic curve (a curve with two inflections) can be represented by including time^3^. The GCA tests the interaction of a condition in the design with linear and quadratic terms on eye movements in the model; in doing that, this analysis can reveal whether the fixation proportion in two conditions differs in the slope or peak. Further time terms (e.g., cubic, quartic) can be included in the model if an effect is expected to show corresponding time-course differences (e.g., fixation proportion is expected to increase, then decrease and increase again in one condition but not in the other). The GCA can also model group or individual differences by including between-subject factors (e.g., age, native language) in the model.

However, if we create power polynomials as we described above, these terms (time, time^2^, time^3^) are highly collinear (i.e., they correlate, with the result that their unique contribution to the results cannot be easily estimated, cf. Glossary). When these independent variables are highly correlated (e.g., if we change the value of time, the value of time^2^ changes accordingly), the model becomes very sensitive to inclusion or exclusion of a highly correlated independent variable. To avoid this problem, the GCA uses so-called orthogonal power polynomials; these are linear transformations of the original polynomials described above and have the advantage that they are less correlated with one another (i.e., their unique effect in the model can be more easily computed).

#### Disadvantages of the GCA

The GCA is not suitable for analysing an interest period in which the fixation proportion stays almost the same throughout the interest period, because there are too few changes in fixations that the time terms (linear, quadratic…) can account for (these terms will then be redundant). For such data, the analyses we introduced in section Analysis of fixation averages for individual interest periods are likely sufficient. The analysis should ideally focus on a relatively short interest period in which an effect is expected to occur, so that the selected interest period does not contain a long time period with only small changes in the fixation proportion. But at the same time, the interest period should not be too short, as it may fail to capture a late effect (cf. section Time window). As such, it has often been used for single-word processing studies, where selecting an interest period is relatively straightforward and the interest period is short. If the time window selection is difficult because it is difficult to predict when an effect of interest is likely to occur based on the study design, the GCA may not be the first choice, and analyses that are less sensitive to the time window selection may be preferred.

**Fig. 4:**
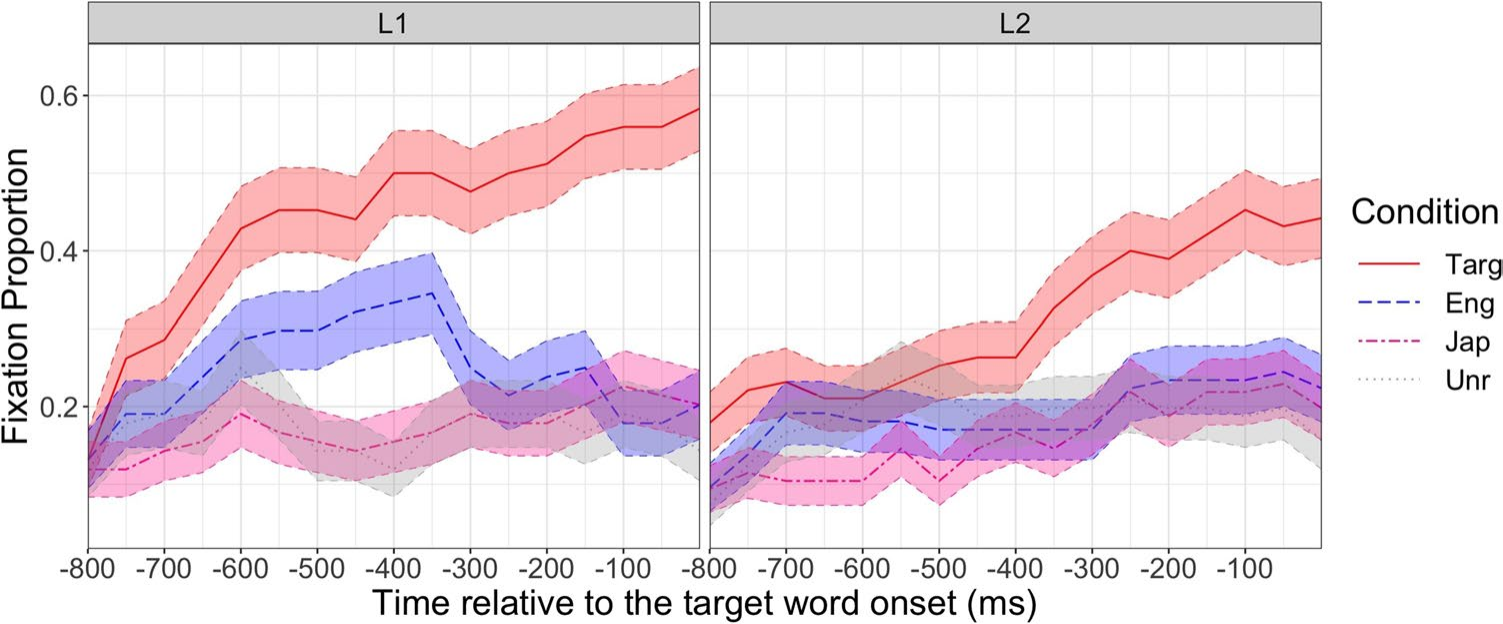
Mean fixation proportion over time for each condition (Targ = target, Eng = English competitor, Jap = Japanese competitor, Unr = unrelated) and group (L1 vs L2). The data are from Ito et al. (2018b). The error bars represent standard errors

We also encourage researchers to define the interest period before data collection, because the choice of the interest period can affect the results (Peelle & Van Engen, 2021). For example, the L1 group’s data from Ito et al. (2018b) in Fig. 4 suggests that the fixation proportion to the English competitor increased and then decreased (i.e., showing a quadratic curve), whereas that to the unrelated object changed very little over time. This difference was captured as a significant effect of English competitor (vs unrelated) condition on the quadratic term (see below for more details). If we had chosen the interest period from −800 ms to −400 ms instead, the fixation proportion to the English competitor only increases, so the effect on the quadratic time term would have disappeared.

The GCA does not account for the autocorrelation of eye-movement data we discussed in section Considerations in applying ANOVA /LME, and including higher-order polynomials (e.g., cubic, quartic terms) may increase the chance of a false positive (cf. Glossary) (Huang & Snedeker, 2020). Thus, researchers should carefully select which polynomials to include in the model. One way to select the necessary polynomials is by assessing the expected time-course change. For example, studies using a phonological competitor (e.g., Allopenna et al., 1998) found that fixation proportion to the mentioned target increased sharply after it was mentioned, whereas fixation proportion to a phonological competitor (an object for which the name phonologically overlaps with the target name, e.g., *cloud* and *clown*) increased less sharply and started to decrease earlier than that to the target. In such a design, including the linear and quadratic terms is likely sufficient to capture the linear increase and the quadratic curve (with one peak), and the cubic term is likely unnecessary. The need for higher-order polynomials can also be evaluated by comparing models with and without a higher-order polynomial (e.g., using the *anova()* function in R). If the model with the higher-order polynomial does not significantly improve the model fit relative to the model without it, the higher-order polynomial is likely unnecessary.

#### Analysis

We demonstrate the GCA using an example data set (IPC_binomfix.txt) from Ito et al. (2018b), which investigated pre-activation of phonological information in L1 and L2 speakers using a phonological competitor design (see section Independent and dependent variables for details). The study had a 4 (condition; target, English competitor, Japanese competitor, unrelated) ×2 (group; L1 vs L2) design. The step-by-step tutorial is in IPC_GCA.html and IPC_GCA.Rmd. This file contains binomially coded fixation (1 = fixated, 0 = not fixated), time bin ID, time relative to the target word onset and other experimental variables (subject ID, item ID, trial number, condition, and language group). Figure 4 plots the mean fixation proportion over time from −800 ms to 0 ms relative to the target word onset. This interest period was selected because the objects appeared on the screen 1000 ms before the target word onset, and the study was designed to test prediction (processes that occur before the target word is mentioned). The onset was then shifted 200 ms forward to account for the 200 ms lag to programme eye movements (Saslow, 1967). A visual inspection of the figure suggests that both L1 and L2 speakers looked at the target object (e.g., a cloud, for the context *The tourists expected rain when the sun went behind the* …), but only the L1 speakers looked at the English competitor (e.g., a clown) before the target word onset. The looks to the Japanese competitor object (e.g., a bear—‘kuma’; target: a cloud—‘kumo’) did not differ from the looks to the unrelated object.

Fixation proportion to the competitor was expected to increase initially and then decrease later. To capture this inflection in the slope, the GCA included a second-order (quadratic) orthogonal polynomial, computed using the *code_poly* function in the *gazeR* package (Geller et al., 2020) using the code below (fix.data is the original data set and dat.stat is a new data set that additionally contains orthogonal polynomials). This code creates new variables called ‘poly1’ and ‘poly2’, each corresponding to first-order (linear) and second-order (quadratic) polynomials. Orthogonal polynomials will be created if you set orthogonal=T (true), and setting draw.poly=T will create a plot showing transformed polynomial predictor values.
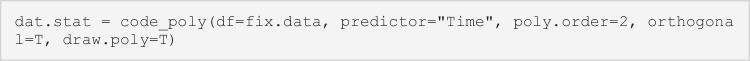


We tested an interaction of condition by language group, effects of condition and language group, and their interactions with time (linear and quadratic terms). The model included by-subject and by-item random intercepts. (In practice, the best model structure should be determined depending on the study design and model fit; cf. Barr, 2013; Barr et al., 2013; Bates et al., 2015; DeBruine & Barr, 2021; Matuschek et al., 2017). The code for running the model is shown below (Count = binomially coded fixation). The results will be stored in gca.cond.lang, which can be called using the *summary* function.
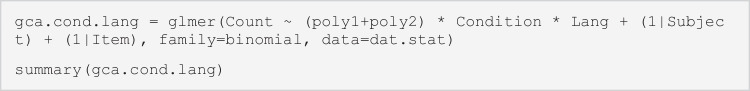


**Fig. 5:**
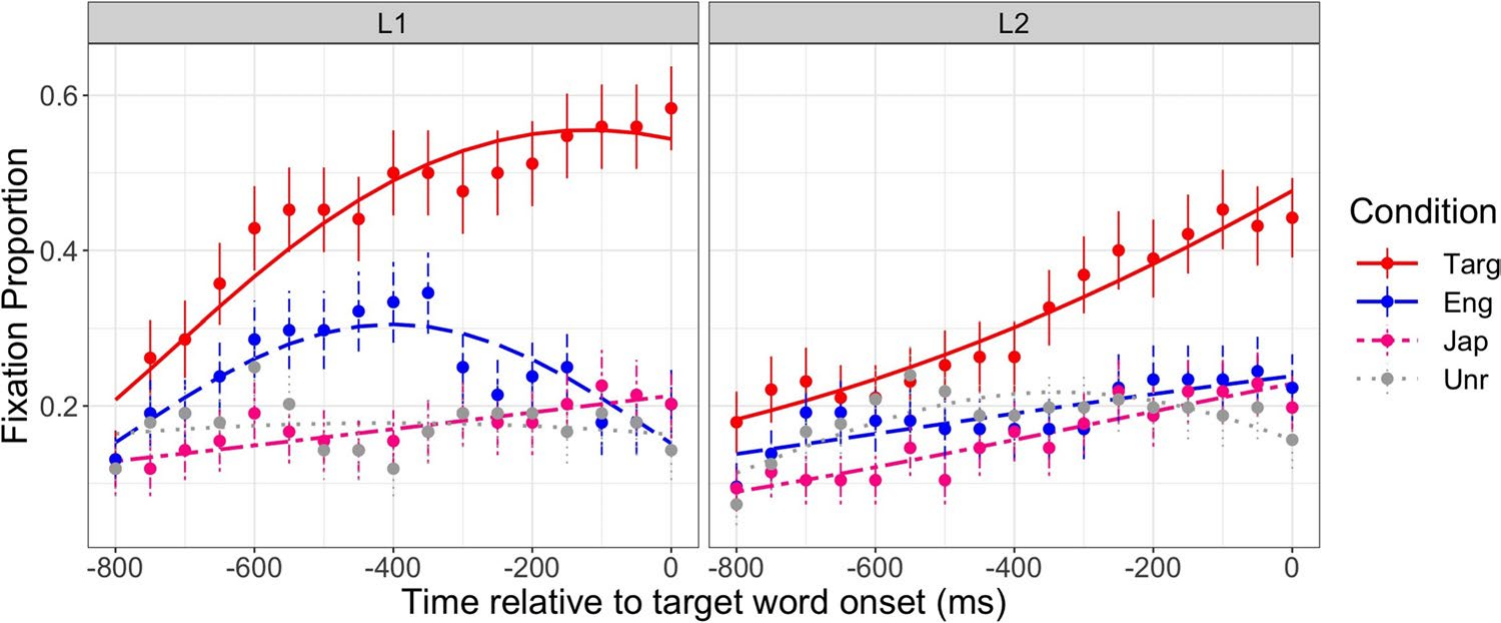
The model fit of the growth curve analysis on the data from Ito et al. (2018b). The points represent the mean, and the error bars around them represent standard errors

The model fit is shown in Fig. 5. We first report overall differences between the conditions indicated by effects on the intercept. The model showed a significant effect of the target vs unrelated condition on the intercept term, *β* = 1.1, *SE* = .065, *z* = 16.9, *p* < .001, suggesting that the target was more likely to be fixated than the unrelated object overall. It also showed a significant effect of the English competitor vs unrelated condition on the intercept term, *β* = .27, *SE* = .068, *z* = 3.9, *p* < .001, suggesting that the English competitor was more likely to be fixated than the unrelated object overall. These effects interacted with the language group, suggesting that these differences were larger in the L1 group than in the L2 group (target vs unrelated, *β* = .35, *SE* = .065, *z* = 5.3, *p* < .001; English competitor vs unrelated, *β* = .22, *SE* = .068, *z* = 3.2, *p* = .001).

Now, we turn to the effects on linear and quadratic terms for interpreting the time-course differences. There was a significant effect of the target vs unrelated condition on the linear term, *β* = 1.8, *SE* = .27, *z* = 6.6, *p* < .001. The effect on the linear term indicates a difference in the slope, so it suggests that the fixation proportion to the target increased more sharply than that to the unrelated object. There was also a significant effect of the Japanese competitor vs unrelated condition on the linear term, *β* = .91, *SE* = .30, *z* = 3.0, *p* = .002, suggesting that the fixation proportion to the Japanese competitor increased more sharply than that to the unrelated object. The model additionally showed an interaction of the target vs unrelated condition by the language group, *β* = −.81, *SE* = .27, *z* = −3.0, *p* = .003, and an interaction of the English competitor vs unrelated condition by the language group, *β* = −.98, *SE* = .29, *z* = −3.4, *p* < .001, on the quadratic term.

To resolve these interactions, we ran the model separately for each language group. The model in the L1 group showed a significant interaction of the English competitor vs unrelated condition on the quadratic term, *β* = −1.1, *SE* = .41, *z* = −2.8, *p* = .005. The effect on the quadratic term captures the difference in the quadratic curve. Specifically, the English competitor condition showed a clear peak around 400 ms before the target word onset, whereas the fixation proportion to the unrelated object stayed relatively flat. The model in the L2 group showed an effect of the target vs unrelated condition, *β* = .97, *SE* = .38, *z* = 2.5, *p* = .01, and an effect of the English competitor vs unrelated condition, *β* = .81, *SE* = .40, *z* = 2.0, *p* = .04, on the quadratic term. These interactions seem to be driven by the unrelated condition, which showed a gentle peak around the centre of the interest period, whereas the fixation proportion to the target increased linearly, and that to the English competitor stayed relatively flat.

These results suggest that both L1 and L2 speakers pre-activated representations of the target word, consistent with the ANOVA/*t*-test results (section ANOVA, *t*-test). The GCA additionally revealed a significant effect of the target (vs unrelated) condition on the linear term in both L1 and L2 groups, and a significant effect of the English competitor (vs unrelated) condition on the quadratic term in the L1 group. The target effect on the linear term suggests that both L1 and L2 speakers were gradually more likely to look at the target as time increased (compared with the unrelated object). The competitor effect on the quadratic term suggests that L1 speakers started to look at the English competitor and then looked away from it. This further suggests that L1 speakers pre-activated phonological information before the target word onset, but the activation of the phonological competitor word decayed quickly.

### Cluster-based permutation analysis (CPA)

The CPA is a non-parametric test that can reveal whether an effect of condition is significant somewhere in a predefined interest period (Maris & Oostenveld, 2007).

#### Advantages of CPA

The CPA can detect an effect of condition while controlling for autocorrelation of eye-movement data. This technique was originally developed for electroencephalography (EEG) or magnetoencephalography (MEG) analysis, and it was later applied to a VWP data analysis (Barr et al., 2014; Hahn et al., 2015; Hirose & Mazuka, 2017). An advantage of the CPA is that it maintains statistical power while controlling for multiple comparisons across many time bins. Thus, it reduces the chance of both type I and type II errors. Moreover, since it is a non-parametric test, the data do not need to meet the assumptions of parametric tests (see section ANOVA, *t*-test).

#### Disadvantages of CPA

One thing that researchers must be aware of in interpreting the results of a CPA is that these data do not reveal the onset, offset or duration of an effect (Maris & Oostenveld, 2007; Sassenhagen & Draschkow, 2019). This is because the *p*-value is derived from cluster-level statistics, so there is no statistical certainty or confidence about a specific time bin in the cluster. Thus, for example, when a cluster in the observed data included time bins from 200 ms to 500 ms relative to a critical word onset, researchers should not interpret this result as the effect starting at 200 ms, ending at 500 ms, or lasting for 300 ms. Instead, the effect should be interpreted as a significant difference between the two conditions. If the time window for the analysis is preselected, a significant result would indicate a significant effect of condition somewhere in the selected time window. For example, a researcher may preselect a time window of 500–800 ms relative to a critical word onset and find a cluster that extended from the beginning to the end of the entire selected time window. This result would suggest that the effect of condition was significant, but it cannot be concluded that there was a significant effect in the entire time window (i.e., that the effect lasted for 300 ms). Another consideration is that the CPA may have reduced power for detecting the smaller (the second largest, the third largest…) clusters, because *p*-values are calculated under the permutation distribution of the maximum (in absolute value) cluster-level statistic (Maris & Oostenveld, 2007).

#### Analysis

The analysis flow of the CPA is as follows.

1. The CPA uses fixation data calculated for small time bins (cf. Glossary). We detect *clusters*—consecutive time bins in which an effect (in the same direction) is significant—and compute a *cluster mass statistic*—typically a sum of all the individual test statistics (e.g., *t*-statistics for *t*-tests) in the cluster. We used the *detect_clusters_by_effect* function in the *clusterperm* package.
2. We create a null hypothesis distribution, which each cluster in the original data set in (1) will be compared against. We used the *cluster_nhds* function in the *clusterperm* package. To do this, we permute the data while respecting the assumption of permutation tests (cf. Glossary) that observation labels are exchangeable under the null hypothesis. For example, if the condition is manipulated within subjects, we shuffle the condition labels within subjects. Between-subject factors should be shuffled between subjects. The *exchangr* package offers several shuffling functions for different manipulations (e.g., *shuffle_each* for within-subject factors, *shuffle_sync* for between-subject factors).
3. We then repeat step (2) and create a large number (1000 or more) of shuffled data sets.
4. On each data set, we repeat step (1) to compute a cluster mass statistic and store the largest cluster mass statistic (in absolute value) for each data set. The distribution of these cluster mass statistics over the shuffled data sets provides a null hypothesis distribution. When steps (3) and (4) are repeated an infinite number of times, the distribution obtained is called a *permutation distribution*, and the corresponding *p*-value obtained at step (5) is called the *permutation p-value*. However, it is practically impossible to repeat this process an infinite number of times, so we obtain a so-called *Monte Carlo estimate*, which can be obtained by repeating this process many times and by comparing the test statistics from the permuted data with the observed test statistic. We need to repeat the process many times because the accuracy of the Monte Carlo estimate increases as the number of permutation processes increases.
5. Finally, we compare each cluster in the original data set with the null hypothesis distribution. The *p*-value is calculated as the proportion of the cluster mass statistics from the null hypothesis distribution from step (4) that resulted in a larger test statistic than that from the original data set. We used the *pvalues* function in the *clusterperm* package. Because this analysis involves permutation, the exact size of cluster(s) and *p*-value may change slightly every time we run the analysis.

To illustrate the process of creating a null hypothesis distribution using an analogy, one can imagine shuffling phonemes between conditions but within the same position (as we do not shuffle across time bins). Taking Altmann and Kamide’s (1999) study, for example, the sentence in the restrictive condition *The boy will eat the cake* is exchangeable with the sentence in the non-restrictive condition *The boy will move the cake* under the null hypothesis. If we exchange phonemes of the verb between the conditions, we obtain sentences such as *The boy will eov/mate the cake* (e.g., /v/ from *move* is exchanged with /t/ from *eat*), *The boy will mat/eove the cake*, *The boy will eav/mote the cake* and so on. As we do not expect differences between the shuffled pairs of sentences (unless all or no phonemes are swapped), we can create a null hypothesis distribution with sufficient shuffling.

Below, we demonstrate the CPA using an example data set (KC_timecourse.txt) from Knoeferle and Crocker (2006). The step-by-step tutorial is in KC_CPA.html and KC_CPA.Rmd. The data file contains binomially coded inspection (1 = inspected, 0 = not inspected), time (sentence onset = 1000 ms), interest area (where the inspection fell) and other experimental variables (subject ID, item ID and condition). For the CPA, we computed the log-ratio indicating an inspection bias towards the agent over the patient using the formula log((Agent inspection+.5)/(Patient inspection+.5)). We ran a by-subject analysis testing whether the log-ratio was significantly different between the MV and RR conditions. The R codes are shown below (the numbers correspond to the steps described above):

(1) Detect clusters:

We used an ANOVA testing an effect of condition on the log-ratio (multiple comparisons were uncorrected because this test is only used for detecting clusters descriptively and not for assessing their statistical significance).
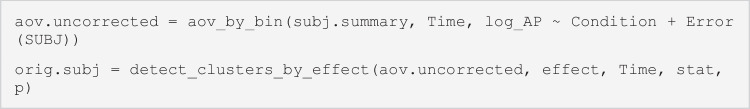


(2–4) Permute the data and create a null hypothesis distribution:

We permuted the data 1000 times. The ANOVA formula passed to the *cluster_nhds* function must be identical to the formula used above (for the bin-by-bin analysis).
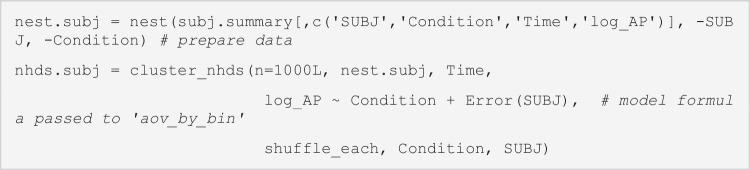


(5) Compare each cluster in the original data set with the null hypothesis distribution and obtain *p*-values (in step (5) cluster significance is assessed):

 We conducted a CPA based on an ANOVA above, but it is also possible to use an LME as a base test. We included an example using the *clusterperm.lmer* function in the *permutes* package (Voeten, 2022) in the script, and below is an example code to run an LME-based CPA. The base LME test included by-subject and by-item random intercepts.
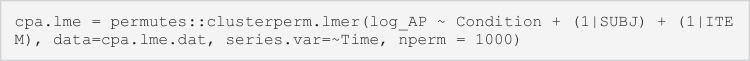


**Fig. 6:**
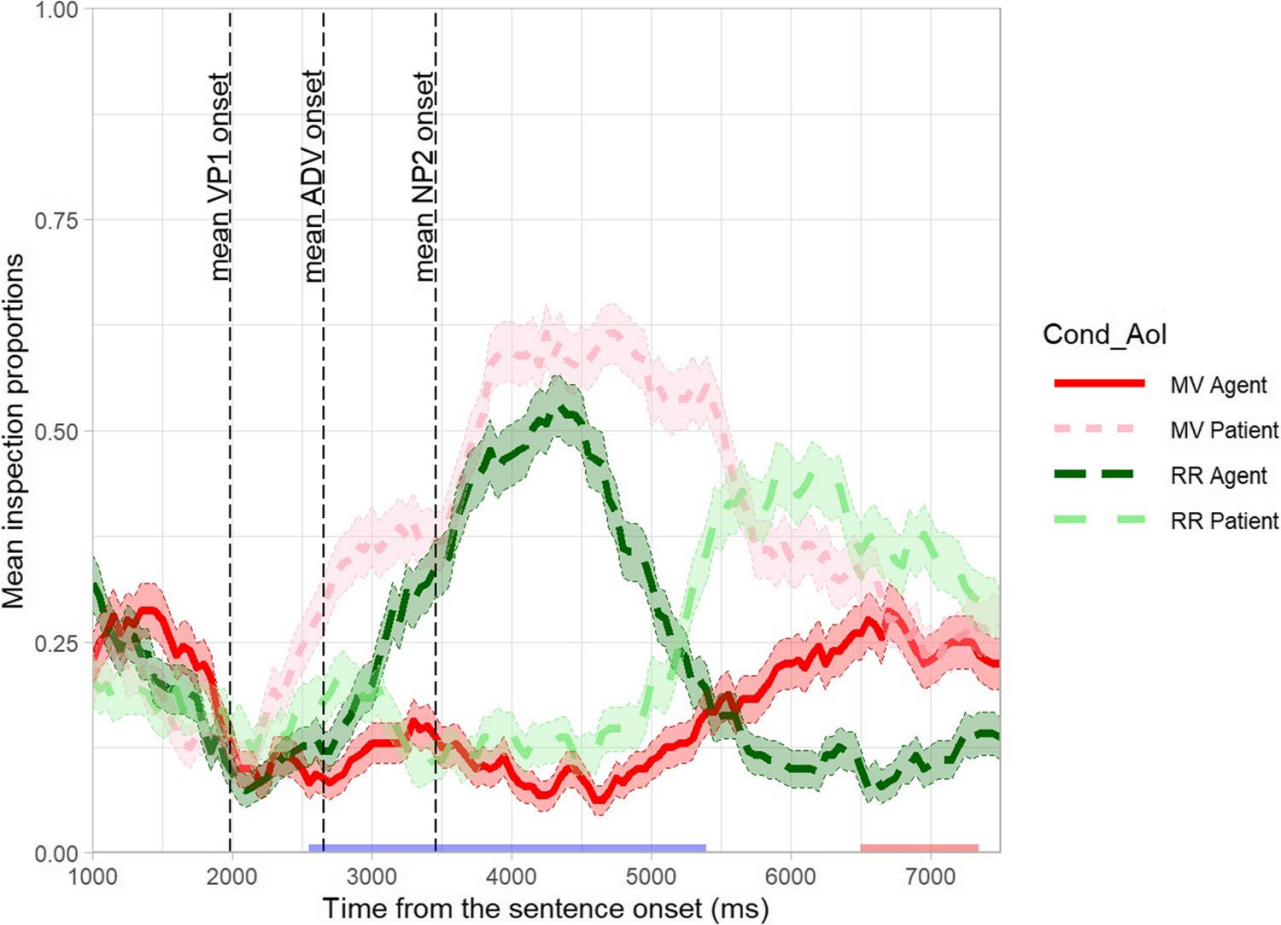
Mean inspection proportion over time for each condition (MV = main verb, RR = reduced relative) and AoI (agent vs patient). The data are from Knoeferle and Crocker (2006). The error bars represent standard errors. The red line at the bottom (*y* = 0) indicates the significant positive cluster, and the blue line indicates the significant negative cluster

Figure 6 plots the time-course graph with the results of the CPA based on ANOVAs at the bottom. A visual inspection of the graph suggests that the patient was inspected more often than the agent in the MV condition whereas the agent was inspected more often than the patient in the RR condition around 2500–5500 ms. This was confirmed by the negative cluster (2550–5400 ms; cluster mass statistic = 3479, *p* < .001), indicating that the log-ratio was significantly more negative in the MV condition than in the RR condition. The opposite pattern is visible towards the end of the time window in Fig. 6, and this was confirmed by the positive cluster (6500–7350 ms; cluster mass statistic = 174, *p* = .03), indicating that the log-ratio was more positive in the MV condition than in the RR condition.

The negative cluster (blue line on *x*-axis) spanning the ADV and NP2 interest periods replicates the results reported in Knoeferle and Crocker (2006), and indicates that participants were more likely to inspect the patient over the agent in the MV condition than in the RR condition. A late time window which covers the positive cluster (red line on *x*-axis) found in the CPA was not analysed in Knoeferle and Crocker (2006). The positive cluster indicates that late in the NP2, participants were more likely to inspect the patient more in the RR condition than in the MV condition.

### Bootstrapped differences of timeseries (BDOTS)

The BDOTS fits a four-parameter logistic or double-Gaussian curve, which is often suitable for VWP time-course data, and estimates when an effect was significant (Oleson et al., 2017; Seedorff et al., 2018).

#### Advantages of BDOTS

The BDOTS can detect time bins where an effect was significant, so it is useful if the research question concerns when an effect starts or how long it lasts. It controls family-wise error that can arise from autocorrelation using a modified Bonferroni correction. A standard Bonferroni correction can make test statistics overly conservative, as it does not take autocorrelation into account. (When a test is repeated in 100 adjacent time bins, the 100 comparisons are not independent but related because of autocorrelation.) The modified Bonferroni correction estimates the autocorrelation between test statistics, and this autocorrelation is used to estimate an adjusted α value. This is less conservative than a standard Bonferroni correction but maintains an overall family-wise error level at a specified α. Additionally, it minimises an influence from researchers’ choices (i.e., the researcher degrees of freedom) because statistical inferences are not made on the direct basis of those choices.

#### Disadvantages of BDOTS

There are some cautions required when fitting a curve. There is the possibility that a specified function does not capture some participants’ curves while capturing others’ very well. Since poorly fit data can affect the statistical results, some measure needs to be taken to deal with this issue. Seedorff et al. (2018) offer some options, such as specifying better starting parameters or relaxing the assumption of autocorrelated errors. The currently available fitting functions (four-parameter logistic or double-Gaussian) in the *bdots* package (Nolte et al., 2021) seem suitable for fitting a curve to a single-word time window, and they may not be able to fit a good curve to a relatively long interest period in which multiple fixation proportion peaks are observed. The BDOTS cannot capture crossed random effects or model correlation (though they may be implemented in the future). The former may be resolved by conducting and combining by-subject and by-item analyses. Another downside is that the time-course estimates obtained from the BDOTS cannot be compared between conditions or groups because we cannot estimate variability across time, though this may be possible if combined with another approach.

#### Analysis

The analysis steps for the BDOTS are as follows. Further descriptions can also be found in the vignettes of the *bdots* package (Nolte et al., 2021).

1. We estimate fitted curves (parameter values and standard errors) for each subject. The *bdots* package currently offers two non-linear functions to do so (a four-parameter logistic and a double-Gaussian). The four-parameter logistic is suitable for a fixation curve for a mentioned object or word, where the fixation proportion typically stays low at the beginning, then increases sharply, and finally reaches the peak and stays high for a while. The double-Gaussian is suitable for a fixation curve for a competitor, where the fixation proportion typically stays low at the beginning, increases, and then decreases after reaching a peak.
2. It is important to inspect and compare the fitted curves and the data at this point to ensure the goodness of fit of the curves.
3. If the curves are fit poorly, we can try refitting the curves.
4. We then draw random samples for each subject’s curve (i.e., bootstrap) and use the definition of the curve to estimate the standard errors of the mean in each time bin for the resampled subject. At each resampling, all the sampled curves are averaged to create a population mean, which provides an estimate of the bootstrapped mean difference and standard error between the groups in each time bin. The function used for curve-fitting is discarded after this step (so statistical inferences are not made based on individual fits).
5. Using the estimated mean difference and standard error, we calculate *t*-statistics and estimate an adjusted α value based on the autocorrelation between test statistics for each time bin.

We attempted to apply the BDOTS to the data set (IPC_fix_50ms_bin.txt) from Ito et al. (2018b), but we could not obtain reasonably well-fitted curves. Thus, our BDOTS tutorial (IPC_BDOTS.html, IPC_BDOTS.Rmd) should not be regarded as a successful application of the BDOTS, but the codes can be applied to a different data set. Below we discuss why we think the curve-fitting did not work for our data. The data file for the tutorial contains fixation proportion (proportion of time spent fixating on the interest area), the number of right-eye samples on the critical interest area, in a blink event or outside the interest areas, and the sum of all samples for each time bin, as well as other experimental variables (subject ID, trial number, item ID, condition and language group). We used the empirical-logit transformed fixation proportion as the dependent variable and tested when the target object attracted more looks in the L1 group than in the L2 group in the interest period from −1000 ms to 1000 ms relative to the target word onset.

- (1) Fit curves:

Here, we used a four-parameter logistics logistic() for the curve-fitting, as we wanted to analyse fixation to a mentioned target. For a double-Gaussian fit, doubleGauss(concave=T) should be used instead. The argument concave=T indicates concave up (concave=F would indicate concave down).

(2) Inspect fitted curves:

We can plot fitted curves using the plot function.
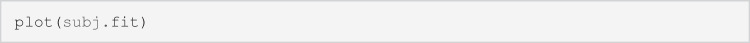


**Fig. 7:**
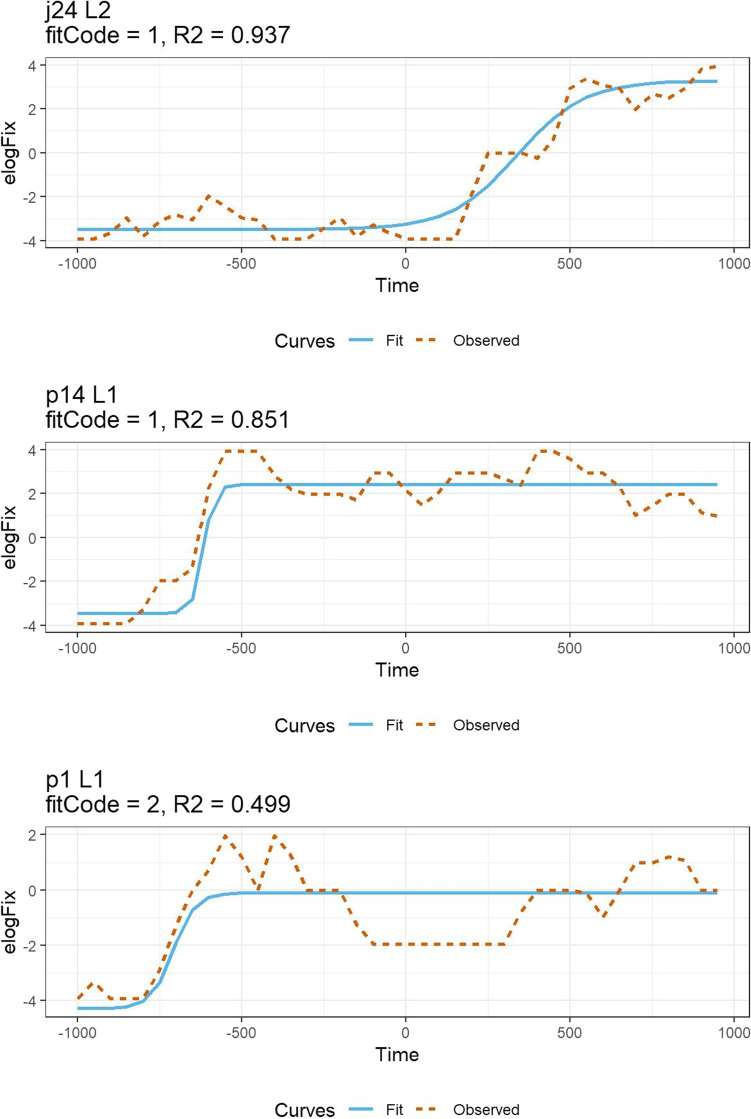
Comparison of fitted curves (solid lines) in BDOTS with the observed data (dashed lines)

Figure 7 shows fitted curves and observed data in the target condition for three participants (j24, p14 and p1) as an example. *R*^2^ can be used as an estimate for goodness of fits. It should ideally be larger than .95 (indicating a good fit), but a value between .8 and .95 is seen as acceptable. The figure also provides “fitCode”, which is 0 for *R*^2^ ≥ .95, 1 for .95 > *R*^2^ ≥ .8, and 2 for *R*^2^ < .8 (indicating a poor fit). As we can see in Fig. 7, the fitted curves were often away from the observed data even when the fits were reasonably good (j24 and p14). When the fit was poor (p1), we can see that the distance between the two lines was much larger (fitted data for all participants are available in the tutorial, IPC_BDOTS.html). An example of well-fitted curves can be found in the vignettes of the *bdots* package (https://cran.rstudio.com/web/packages/bdots/vignettes/bdots.html). It is not recommended to proceed with the analysis when most of the curves show a poor fit, as in our data set, but we present the analysis simply for didactic purposes.

(3) Re-fitting:

We can use the bdotsRefit function to re-fit the curves.
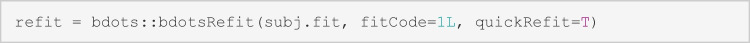


(4–5) Bootstrapping:

The code below will test a difference between the L1 and L2 groups.
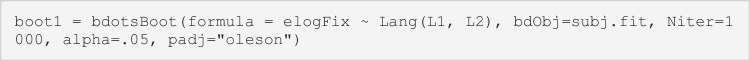


We can get the model summary and plot using the codes below.
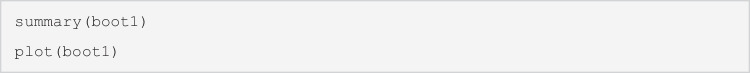


**Fig. 8:**
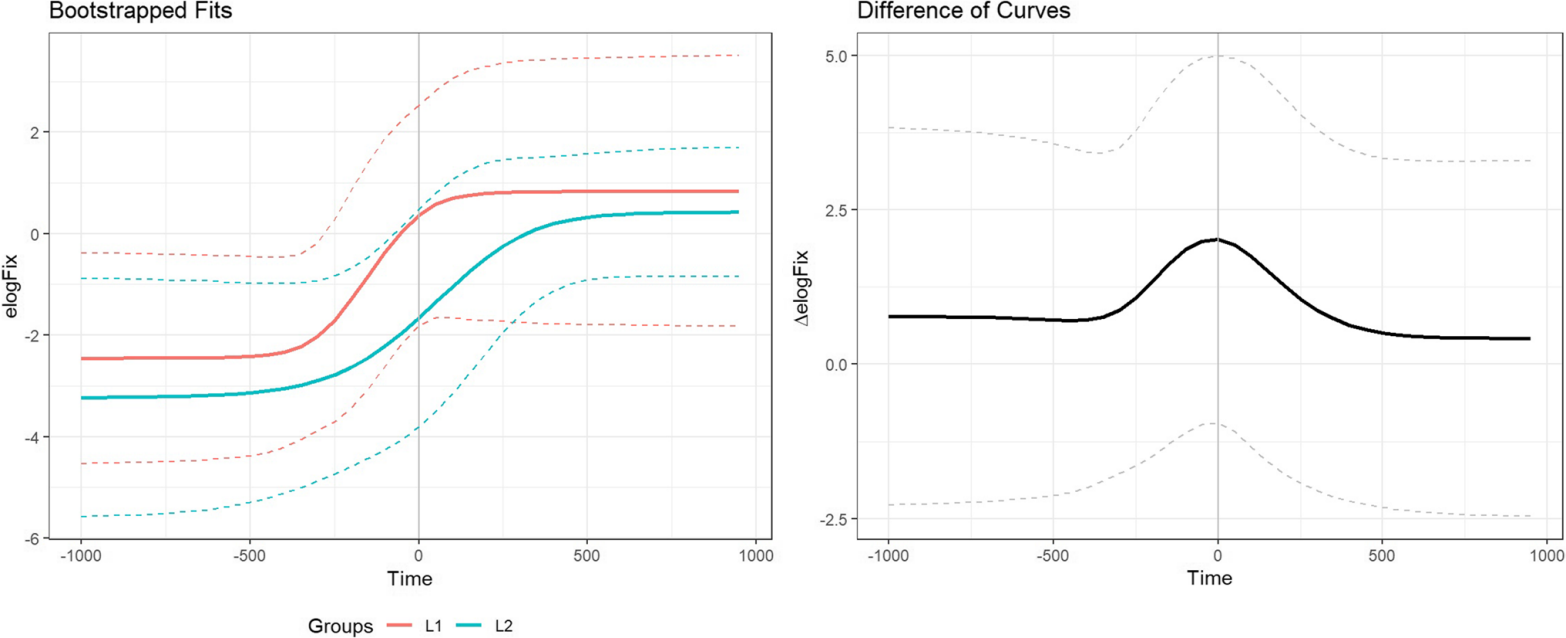
The results of the BDOTS on Ito et al. (2018b)

As Fig. 8 suggests, the BDOTS did not detect any significant time bins for this data set, inconsistent with the visual inspection of the data (cf. Fig. 4 or Fig. 10) and the results from the ANOVA (section ANOVA, *t*-test) and the GCA (section Growth curve analysis (GCA)). This is expected, because if curves for individual participants are fit poorly, the standard deviation estimates for the parameters can be extremely large, as we can see in Fig. 8. Thus, it is crucial for this analysis to visualise the fitted curves and ensure the goodness of the fits.

Why couldn’t the BDOTS fit good curves for our data? We think one possibility is that the number of observations per condition per participant was too small for the curves from the empirical data to be smooth. Ito et al. (2018b) used 16 critical trials (with four observations per condition per participant), whereas the number of critical trials in studies that successfully used the BDOTS is much larger (336 trials in McMurray et al., 2019; 480 trials in Sarrett et al., 2022; 384 trials in Hendrickson et al., 2021; 216 trials in Kapnoula & Samuel, 2019). In principle, we do not need to use a four-parameter logistic or double-Gaussian function, and any curve-fitting function can be used for the BDOTS (e.g., polynomials we used for the GCA). It is also possible to fit different functions for different participants. However, the problem here does not seem to be that each participant showed a different curve, but that the data averaged for each subject tended to show non-smooth curves. Thus, by-participant averaged fixation curves need to be suitable for curve-fitting, so that they can be fitted using available curve-fitting functions.

### Generalised additive mixed modelling (GAMM)

The GAMM is a regression model that can model non-linear time-course data and estimate when an effect of interest occurred (Porretta et al., 2018; Wieling, 2018; Wood, 2011).

#### Advantages of GAMM

Linear models such as LMEs discussed above assume a linear relationship between independent variables and the dependent variable. The GAMM does not have that assumption, so it can model both linear and non-linear relationships. In that sense, it is similar to the GCA but unlike the GCA, the GAMM does not use polynomials (but thin plate regression splines, see below). This is advantageous, as time-course data from a VWP experiment often have a non-linear form. The GAMM guards against false-positive errors in two ways. First, it can account for autocorrelation by including an AR1 autocorrelation parameter in the model and adjusting the confidence of the estimates accordingly (Porretta et al., 2018). In GAMM using the *bam* function (*mgcv* package), this autocorrelation parameter is obtained by computing the cross-correlation between data at a first time point and data at the next time point (1 in AR1 indicates that the correlation is computed using a time lag of 1, i.e., correlation between adjacent time points). AR1 is a simple model (in that it depends only on the closest previous time point) but it can alleviate the autocorrelation problem sufficiently in most cases (Wieling, 2018). Different autocorrelation parameters (e.g., partial autocorrelation) can be used in different functions (e.g., the *gamm* function in the *mgcv* package). Second, the GAMM models the non-linear curve by using thin plate regression splines, which combine increasingly complex non-linear basis functions (Wood, 2003), as they are flexible in fitting complex non-linear curves. In this way, the data guide the functional form, preventing the risk of overfitting or underfitting.

#### Disadvantages of GAMM

**Fig. 9:**
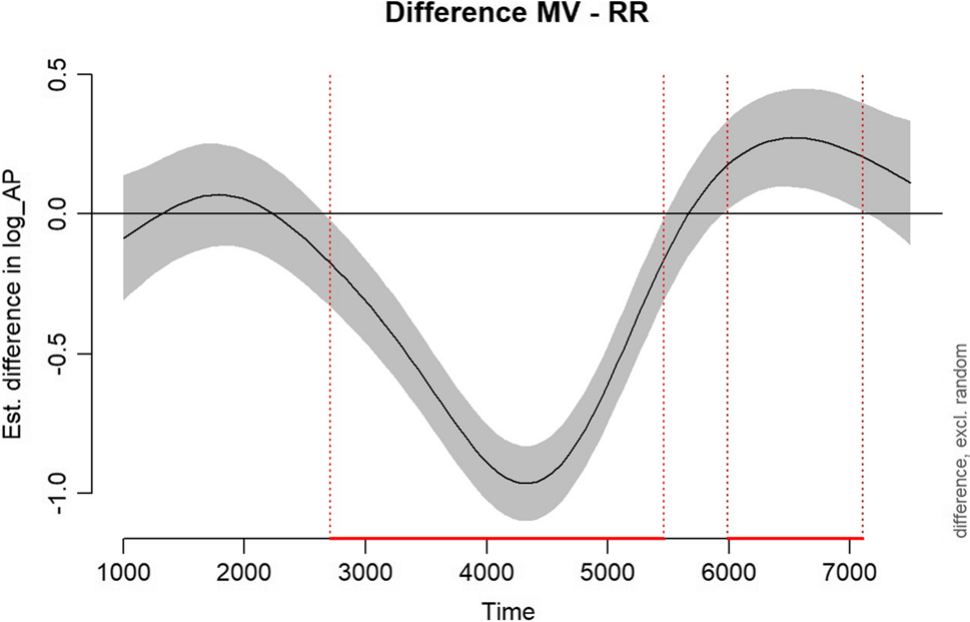
Log-ratio difference plot for MV vs RR conditions. The shaded area indicates 95% confidence intervals. The red lines at the bottom indicate time bins in which the difference between the conditions was significant

While the GAMM can estimate when an effect started to occur and how long it lasted, it cannot statistically compare the onset or duration of an effect between conditions or groups (i.e., it cannot test whether an effect started significantly earlier in one condition than in another) because it does not provide a measure of variability (e.g., confidence interval) for the timing of the effect. Another downside is that if binomially coded data (cf. section Independent and dependent variables) are used as a dependent variable, the GAMM cannot account for autocorrelated errors because binomial distribution cannot be used for that (unlike Gaussian distribution) (Porretta et al., 2018). To account for autocorrelation, researchers can use empirical-logit transformed fixation proportion (cf. section ANOVA, *t*-test). While the GAMM can model complex time-course data, the model parameters (i.e., coefficients) are not easily interpretable. For example, a significant effect of time or its interaction with an experimental condition does not tell us the onset or duration of an effect. The GAMM estimates the onset or offset of an effect by computing a difference plot, as we demonstrate below (Fig. 9).

#### Analysis

We used the example data set (KC_timecourse.txt) from Knoeferle and Crocker (2006) for the GAMM tutorial (KC_GAMM.html, KC_GAMM.Rmd). The data file contains binomially coded inspection (1 = inspected, 0 = not inspected), time (sentence onset = 1000 ms), interest area (where the inspection fell) and other experimental variables (subject ID, item ID, and condition). We used the log-ratio indicating an inspection bias towards the agent over the patient using the formula log((Agent inspection+.5)/(Patient inspection+.5)) as our dependent variable.

We first ran a ‘base’ model to determine an appropriate value for the AR1 correlation parameter (the parameter to account for autocorrelated residuals). In GAMM, the model formula resembles the formula for a generalised linear model or LME (*lme4* package), but the difference is that smooth terms can be added to the GAMM formula. In addition, random effects are not defined in the same way as in LME. s(Time, by=Condition) specifies a random intercept for time, and by=Condition is used to model potentially different trends over time for different conditions. s(Time, SUBJ, by=Condition, bs="fs", m=1) specifies a random smooth (a non-linear random effect; “fs” = factor smooth), and by=Condition here is used to account for individual variation (a random slope for condition). Note that if factor smooths are added, these already incorporate random intercept and random slope effects, so the model does not have additional specifications for them (cf. Wieling, 2018). If random intercepts or linear random slopes are desired instead of a random smooth, they can be included as s(SUBJ, bs=”re”) (a by-subject random intercept; “re” = random effect) or s(SUBJ, Condition, bs=”re”) (a by-subject random slope for condition).
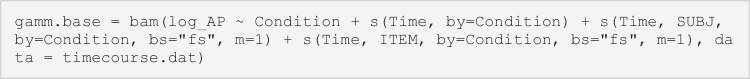


The start_value_rho function provides a value we can use as the AR1 correlation parameter. We assigned this value to AR1.val to include this in the main model.
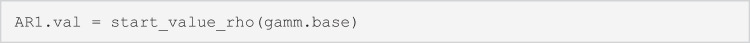


The syntax for the model including the AR1 correlation parameter is shown below. We additionally need to include a logical vector indicating the starting time point for each trial. In our data, the Is_start column contains TRUE if the current time bin is the starting time point (= 1000 ms), and FALSE otherwise.

 We can check whether accounting for autocorrelation improved the model fit by plotting autocorrelation and by comparing the ‘base’ model with the model with the AR1 correlation parameter using the compareML function.

 We can plot a difference curve to estimate the time when the effect of condition (MV vs RR) occurred.
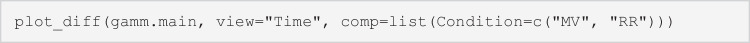


Figure 9 plots the difference curves (MV minus RR) (see Fig. 6 for the means for each condition). The results show that the log-ratio was more negative in the MV condition than in the RR condition from 2707 ms to 5465 ms, and it was more positive in the MV condition than in the RR condition from 5990 ms to 7106 ms. To enable a meaningful interpretation of these differences in relation to events in the utterance, the relation of these time points (e.g., 2707 ms) to average word onsets and offsets of the target sentences must be determined (see Fig. 6 for values).

### Divergence point analysis (DPA)

The DPA is a non-parametric test that can estimate when two fixation proportion curves start to diverge from one another (Stone et al., 2020).

#### Advantages of DPA

The DPA can estimate the onset of an effect and a confidence interval. Thus, it is possible to statistically test differences in the onset (divergence point) between conditions or groups, overcoming the shortcomings of the BDOTS or the GAMM. It can also yield a *p*-value by comparing the distribution of the divergence points and the null distribution. This analysis overcomes the multiple comparisons problem and controls for autocorrelation of the eye-movement data. Like the CPA, the DPA is a non-parametric test, so the data need not meet the assumptions of a parametric test (see section ANOVA, *t*-test). The DPA has further been adapted to incorporate a Bayesian analysis, which allows researchers to quantify evidence for an alternative or null hypothesis using Bayes factors (Stone et al., 2021).

#### Disadvantages of DPA

While this analysis can test when an effect started to occur, it does not test how long the effect lasted. It also cannot test multiple divergence points (it only detects the first divergence point). Another important point is that this analysis assumes that the effect is present. Thus, if researchers want to test whether there is an effect and when the effect started to occur, a separate analysis is needed to establish whether the effect is significant^6^.

#### Analysis

The analysis flow of the DPA is as follows.

1. The DPA uses fixation data calculated for small time bins (cf. Glossary). We run a statistical test for each time bin (e.g., *t*-test, linear mixed-effects model). In non-parametric tests, *t*-tests are often preferred over mixed-effects models, as they are computationally lighter and have no convergence issues.
2. We establish a divergence point by taking the first of multiple consecutive time bins for which the effect of interest was significant in the same direction. The minimum number of bins in which the effect must occur should be determined depending on the research question and the study design. If it is hypothesised that a fixation bias should sustain for at least 200 ms, and the size of each time bin is 20 ms, then 10 consecutive time bins will be a suitable threshold.
3. Step (2) will yield a single divergence point. To estimate what the distribution of the divergence points would be if the experiment is repeated many times, we create an alternative data set by bootstrapping (resampling) the original data set by participant, time bin and condition/object.
4. We then repeat steps (1) and (2) on the alternative data set to establish the divergence point for the resampled data set.
5. We repeat (3) and (4) many (1000–2000) times to generate the bootstrap distribution of the divergence points. We can then calculate the mean of the divergence point and the confidence interval.
6. If we want to test whether the divergence point is different between two conditions or groups, we will compute a *p*-value by comparing (5) to a distribution of divergence point that could be expected under the null hypothesis. The *p*-value is the proportion of samples from the null distribution that are larger than the observed difference in the divergence point in the empirical data. Because this analysis involves bootstrapping, the exact onset time and *p*-value may change slightly every time we run the analysis.

In this paper, we apply the DPA to the data set (IPC_fix_50ms_bin.txt) from Ito et al. (2018b). The step-by-step tutorial based on the script by Stone et al. (2020) is in IPC_DPA.html, IPC_DPA.Rmd. The data file contains fixation proportion (proportion of time spent fixating on the interest area), the number of right-eye samples on the critical interest area, in a blink event, or outside the interest areas, and the sum of all samples for each time bin. It additionally contains other experimental variables (subject ID, trial number, item ID, condition, and language group). We used the empirical logit (cf. section ANOVA, *t*-test) as the dependent variable and tested when the target object started to attract more looks than the unrelated object in L1 and L2 groups. We ran the DPA in the interest period from −800 ms to 1000 ms relative to the target word onset. The initial 200 ms was not included because the data did not contain many observations (as visible from Fig. 7, and as expected because the objects appeared on-screen 1000 ms before the target word onset). The R codes corresponding to each step above are shown below:

(1) A statistical test for each bin:

We used a *t*-test testing an effect of condition on the empirical logit (elogFixM). The example code below is for the L1 group, and this stores *t*-values in test_g1.

 (2) Establish a (single) divergence point:

The code below is only for the L1 group. In this analysis, we took the first of four consecutive bins for which the effect of condition was significant in the same direction (when the target attracted significantly more fixations than the unrelated object). As our bin size was 50 ms, the analysis assumed that a fixation should sustain for at least 200 ms (4 bins × 50 ms). The codes (1–2) are embedded in a function boot_L1L2 to repeat these steps for the bootstrapped data set.

 (3–5) Bootstrap the data and obtain divergence points for many bootstrapped data sets:

In this example, we set the number of iterations for the bootstrap to 1000.

 The code below bootstraps the original data div.dat 1000 times (to obtain 1000 divergence points).

 (6) Calculate a *p*-value:

To create a null distribution expected under the null hypothesis, we randomly assign group labels (if we want to test a difference between the groups). The code below can be embedded in the boot function to shuffle the group labels.

 We then ran a bootstrap function similar to the one above but using the function that contained the group-shuffling code above (boot_L1L2_pval).

 We can then compute the proportion of samples from the null distribution that are larger than the observed difference in the divergence point in the empirical data (= *p*-value).
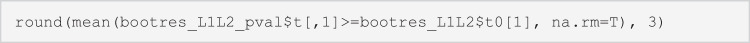


**Fig. 10:**
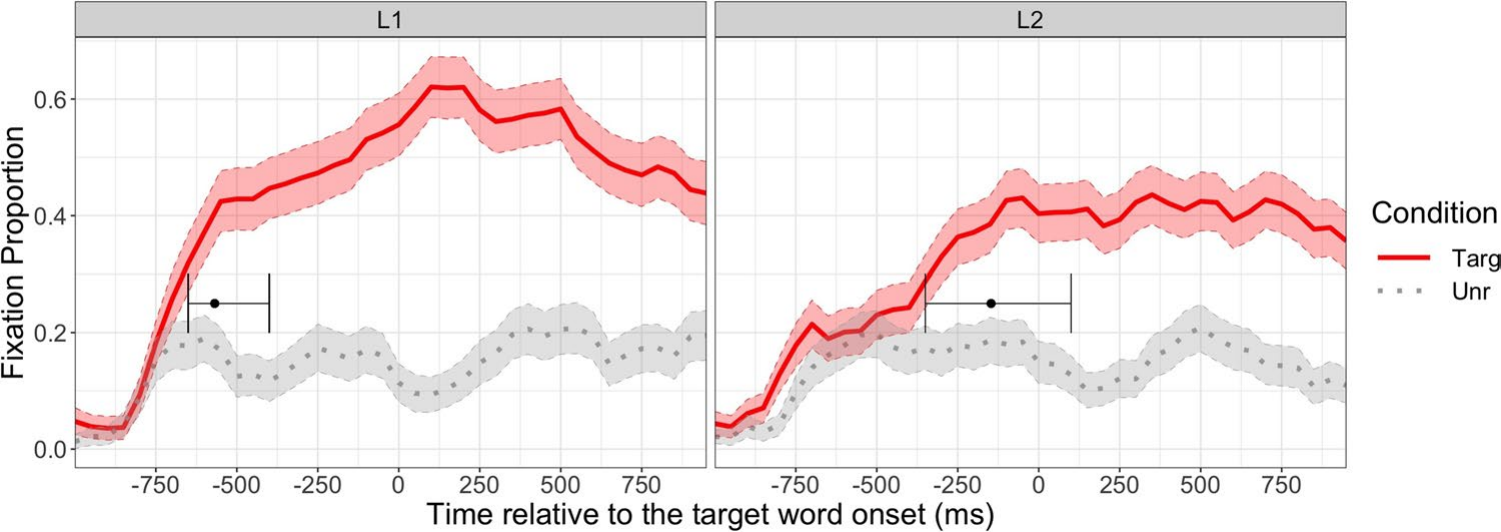
Mean fixation proportion over time for the target (Targ) and unrelated (Unr) conditions for each group (L1 vs L2). The data are from Ito et al. (2018b). The error bars represent standard errors. The divergence point and its confidence interval are plotted as the black dot and black horizontal error bar for each group

The DPA showed that the mean divergence point relative to the target word onset was −574 ms, 95% CI = [−650, −450] in the L1 group, and −155 ms, 95% CI = [−350, 100] in the L2 group (cf. Fig. 10). Because the confidence intervals from these two groups do not overlap, the results suggest that L1 speakers started to look at the target object over the unrelated object significantly earlier than L2 speakers. The DPA shows that the divergence point was 419 ms earlier in the L1 group than in the L2 group, *p* = .02, suggesting that L1 speakers predicted representations of the target word more quickly than L2 speakers. Ito et al. (2018b) used the GCA, so it was not possible to determine when the target started to attract more fixations than the unrelated object, and whether it did so earlier in the L1 group than in the L2 group.

## Summary

In this paper, we have described aspects of the design, stimuli, their timing, and characteristics (section Design, visual and speech stimuli, timing, interest periods and task) of the VWP. We applied these characteristics to an informative description of two example experiments (section Descriptions of our data sets). In section Analysis of fixation averages for individual interest periods, we presented methods for analysing differences in averaged fixation proportion or log-ratio of inspections in individual interest periods (often predefined) in a VWP study. Researchers want to select an appropriate analysis based on the study design and research question, considering the advantages and disadvantages of each method (Fig. 3). If the research question does not concern the precise temporal emergence of an effect of interest and if there is little fixation proportion change (e.g., first increase then decrease) over time, a *t*-test/ANOVA or an LME is probably sufficient for testing an overall difference between conditions or participant groups in a specific interest period. If the data do not include many extreme values (proportions close to 0 or 1), a *t*-test or ANOVA seems to show reasonably similar results to an LME (Stone et al., 2020). An LME would generally be more sensitive and hence more suitable when there is large variability across participants or items (e.g., some participants show a large effect, while others show a much smaller or opposite effect), as it can take that sort of variability into account. It is also suitable when there are many missing values or when the design is not balanced because it can be used straightforwardly for unbalanced data with fewer observations in one condition than another. Note, however, that there are ways of analysing dependent variables with missing values using an ANOVA (e.g., van Ginkel & Kroonenberg, 2014).

Section The temporal emergence of an effect deals with eye-movement analysis when the research question concerns the temporal emergence of an effect. In this case, the GCA, the CPA, the BDOTS, the GAMM or the DPA would be particularly suitable. The GCA is suitable for testing differences in fixation proportion changes over time across conditions or participant groups. The CPA is suitable for testing differences between two conditions or groups over multiple time bins while controlling for multiple comparisons and autocorrelation. The BDOTS is suitable for testing when an effect occurred and when fixation proportion curves averaged for each participant can be explained well with available curve-fitting functions. Typical fixation patterns for a mentioned target or for a competitor object can be modelled using available functions, so it is well suited for a single-word processing study. The GAMM is also suitable for testing a temporal emergence of an effect, and it is good at modelling relatively complex non-linear curves. The DPA is suitable for estimating the onset of an effect and statistically comparing it between two conditions or groups.

## Footnotes

1 Parametric tests have been widely used in psycholinguistic research, and data entered in a parametric test must fulfil specific assumptions; see Jaeger (2008) for a discussion of problems when analysing binary data with parametric analyses (see also Donnelly & Verkuilen, 2017).

2 Inferential analyses are analyses that examine whether an outcome measure is likely a chance result or not. They can, for instance, assess how likely it is that the observed difference in fixation duration (across participants) for one vs another condition (a systematic manipulation by the experimenter) would have been as large as the one observed if the assumption of no difference between the conditions were true (the so-called null hypothesis). If it is very unlikely (e.g., < .05 probability) that a duration difference as large as the one observed would have been found by chance, we can be relatively certain, that this difference is not random variability but systematic (perhaps due to something the researcher did—a manipulation).

3 In an experimental design, researchers specify something called an ‘experimental manipulation’, meaning they introduce different systematic changes. For instance, we could add an adjective before a noun like *woman.* That adjective could either match (e.g., *brunette*) or mismatch (*blonde*) the hair colour of the woman referenced by *woman*. If listeners rapidly rely on such systematic manipulations like a matching adjective, they may start to shift their eye gaze to the brunette woman earlier when the adjective matches than when it mismatches.

4 An alternative would be a ‘between-subjects’ design in which one group of participants is exposed to one level/condition of a manipulated variable while another group of participants is exposed to another level/condition of a manipulated variable. Within-subject designs are often preferred in psycholinguistic and other experiments since they result in less variability associated with the comparison of levels. If you have a one-factor two-level design as in Altmann and Kamide (1999), and 24 critical stimuli, then one participant is exposed to 12 trials of the restrictive verb level and to 12 trials of the non-restrictive verb level in a within-subject design. With a within-subject allocation, differences in eye-gaze between the two verb levels cannot result from differences between participants. By contrast, if one group of participants were exposed to the restrictive verb items and another group to the non-restrictive verb items (as would be the case in a between-subjects design), then differences in participants’ eye movements between the two levels would be confounded with other differences between the participants (e.g., in age, intelligence or language ability).

5 Dan Mirman shares his analyses with empirical logit as proposed by Barr (2008) and with flattened logistic regression as proposed by Donnelly and Verkuilen (2017) in his blog: http://mindingthebrain.blogspot.com/2016/12/flattened-logistic-regression-vs.html

6 Stone et al. (2020) provide data and an analysis tutorial for the DPA at https://osf.io/exbmk/. Their tutorial includes a comparison with other methods of correcting multiple comparisons (Bonferroni correction and false-discovery rate control) as well as results from uncorrected multiple comparisons. They additionally demonstrate autocorrelation in their data.
